# Multi-Season Regional Analysis of Multi-Species Occupancy: Implications for Bird Conservation in Agricultural Lands in East-Central Argentina

**DOI:** 10.1371/journal.pone.0130874

**Published:** 2015-06-18

**Authors:** Andrea Paula Goijman, Michael. J. Conroy, Jaime Nicolás Bernardos, María Elena Zaccagnini

**Affiliations:** 1 Instituto de Recursos Biológicos, CIRN, Instituto Nacional de Tecnología Agropecuaria (INTA), Hurlingham, Buenos Aires, Argentina; 2 D.B. Warnell School of Forestry and Natural Resources, University of Georgia, Athens, GA United States of America; 3 EEA Ing. Agr. Guillermo Covas, Instituto Nacional de Tecnología Agropecuaria (INTA), Anguil, La Pampa, Argentina; University of Bern, SWITZERLAND

## Abstract

Rapid expansion and intensification of agriculture create challenges for the conservation of biodiversity and associated ecosystem services. In Argentina, the total row crop planted area has increased in recent decades with the expansion of soybean cultivation, homogenizing the landscape. In 2003 we started the first long-term, large-scale bird monitoring program in agroecosystems of central Argentina, in portions of the Pampas and Espinal ecoregions. Using data from this program, we evaluated the effect of land use and cover extent on birds between 2003-2012, accounting for imperfect detection probabilities using a Bayesian hierarchical, multi-species and multi-season occupancy model. We tested predictions that species diversity is positively related to habitat heterogeneity, which in intensified agroecosystems is thought to be mediated by food availability; thus the extent of land use and cover is predicted to affect foraging guilds differently. We also infer about ecosystem services provisioning and inform management recommendations for conservation of birds. Overall our results support the predictions. Although many species within each guild responded differently to land use and native forest cover, we identified generalities for most trophic guilds. For example, granivorous gleaners, ground insectivores and omnivores responded negatively to high proportions of soybean, while insectivore gleaners and aerial foragers seemed more tolerant. Habitat heterogeneity would likely benefit most species in an intensified agroecosystem, and can be achieved with a diversity of crops, pastures, and natural areas within the landscape. Although most studied species are insectivores, potentially beneficial for pest control, some guilds such as ground insectivores are poorly represented, suggesting that agricultural intensification reduces ecological functions, which may be recovered through management. Continuation of the bird monitoring program will allow us to continue to inform for conservation of birds in agroecosystems, identify research needed to reduce key uncertainties, and anticipate the effects of changes in agriculture in central Argentina.

## Introduction

An important challenge for conservation is to conserve biodiversity, and its associated ecosystem services, in the face of rapid changes in land use and agricultural intensification in response to a growing demand for food supply [1,2]. Agricultural landscapes deliver planned services like crop or fuel production, but maintenance of biodiversity also provides other associated ecosystem services. However, the intensification of agriculture alters the suitability and extent of habitats for wild birds, and changes community assemblages [3,4]. Well-informed regional solutions are needed to properly balance agriculture intensification and reduce impacts to biodiversity.

Bird communities are comparatively easy to monitor and quantify, and are responsive to environmental perturbations, and thus are an ideal system for evaluating the effects of environment changes and resource availability. Because different species have specific habitat requirements, they respond differently to perturbations, and thus responses to environmental change can be anticipated to be heterogeneous. Some species provide ecosystem services to agriculture (e.g. pest control, pollination, seed dispersal) [5,6], while others are considered pests; in either case, these attributes can be used to classify species into functional groups [7]. Within these functional groups, birds can be identified by guilds that exploit resources in a similar way, and thus would be predicted to respond similarly to habitat changes [6,7]. Classifying birds in guilds could be useful in order to make inferences; however, grouping prior to any analysis could mask individual species responses, biasing conclusions [8,9]. In this study, we are interested in the effects of agriculture on birds and the potential provisioning of ecosystem services in the Pampas grassland and Espinal forests ecoregions in Argentina, where agricultural intensification and expansion have been evident. To assess these effects, it is essential to evaluate the responses of guilds, considering at the same time species-specific responses that could bias conclusions if they are ignored.

In Argentina, the total area planted in row crops has increased rapidly in recent decades, coinciding with the expansion of soybean cultivation introduced in the mid 1970’s [10,11]. The expansion of soybean was facilitated by deforestation and replacement of natural systems, land use intensification, and changes in technologies, all which led to a homogenization of the landscape [10,12]. For example, planted area of soybean increased by more than 400% from 1985 to 2011, making Argentina one of the major soybean exporting countries in the world [11,13]. Agricultural intensification has been evident in the Pampas grassland and Espinal forests ecoregions, where croplands replaced the original grasslands, forests, and pastures for cattle grazing; and soybean area represents more than 50% of Argentine agriculture [11,12]. This rapidly changing scenario could have adverse impacts on biodiversity, and thus of concern to conservation. Many interlinked processes are altered by agricultural intensification and expansion, hindering the identification of a single factor that affects bird communities. For example, in areas with intensified agriculture, the size of the fields tends to be larger, the use of agrochemicals more intense and the landscape is more fragmented than less intensified areas [1,3,4,14]. At a large scale, these processes ultimately affect species by generating loss of high quality habitat for refuge, feeding, and breeding, replaced by a higher proportion of croplands [15,16]. On the other hand, species that perceive the landscape at finer scales could still benefit from local spatial heterogeneity provided by natural vegetation in field borders or roadsides [17,18].

Long-term bird monitoring data exist in many developed countries in Europe and North America, and have been used to evaluate trends and detect declines of birds [19,20]. Monitoring is usually conducted by volunteers, and thus is subject to error and variation, which must be either reduced by design, or controlled for by appropriate statistical analysis to avoid erroneous inferences. In 2003, we initiated a long-term, large-scale monitoring program (Bird Monitoring in Argentina; BMA) in the Pampas and a portion of the Espinal ecoregions in central Argentina which, to the best of our knowledge, is the first long-term regional bird monitoring program in agroecosystems of the southern Latin America [21,22]. To date, detection/non detection data exist for 263 bird species, over 10 years. Unlike other regional bird monitoring programs, BMA is carried out by hired professional birdwatchers following a pre-established methodology, minimizing biases associated with misidentification of species and survey effort. Nevertheless, we recognize that our detection/ non-detection data represent an incomplete sample of actual occurrence, given that detection probabilities likely variable among species, and over space due to factors beyond the control of our design. We address these issues by means of explanatory variables and statistical models discussed in detail below.

There are several studies reporting the responses of birds to land use in these ecoregions, but there are still gaps of information about the effects of agriculture [22]. Most studies are short term or involve selected species [23–25]. To understand the effects of this regional scale process on birds, it is necessary to include longer term, regional studies. However, many of these studies assume perfect detection of the species of interest [23–25]. In other studies using our BMA data, Gavier-Pizarro et al. [26] accounted for imperfect detection using Distance Sampling techniques, on a selected group of species, while Zaccagnini [27] and Thompson [28]–in a more general analyses than the one we implemented in our study, grouping species in three functional groups and pooling all 30 points of every route—incorporated imperfect detection probabilities using occupancy. Occupancy (detection/ non-detection) is a data structure that can be readily gathered for many species using an appropriate design over large spatial extents. It requires appropriate statistical models to account for imperfect and heterogeneous detection probabilities [29–31].

We used multi-season occupancy models to evaluate the effect of land use and land cover (henceforth ‘LULC’) extent on multiple species of birds, using the BMA over 10 years (2003–2012). Under this approach, multiple species are linked together within a hierarchical (or multi-level) model, allowing for a more efficient use of data, and increased precision of occupancy estimates [32–36]. As previously noted, the interactions between birds and the agricultural environment involve several interrelated factors. In this study we specifically evaluated the effect of LULC on the occupancy of birds at the landscape scale over time. Since the BMA is carried out at regional level, we control the variability in occupancy given the geographic location, which may be related to factors operating on a large scale, such as climate and the original geographic distribution of the species. Our study tests the prediction that species diversity is positively affected by habitat heterogeneity and complexity of vegetation structure [1,24,37,38]. Depending on particular characteristics of each species (e.g. size, feeding habits), this relationship can hold for some guilds at a local vegetation or landscape scale [39]. Several authors suggest that in intensified agroecosystems this relationship is probably mediated by the availability of food for birds [15,16,40]. We hypothesized that, because birds may be affected by the availability of food resources, the proportion of LULC in an agricultural landscape affects foraging guilds differently. Within those, it could also affect each species differently, since not two species share the exact same foraging strategies and habitat requirements [41–43].

Because species may respond independently to LULC, predictions of foraging guilds’ responses can be difficult, but we suggest several general principles of the patterns. First, we predict that, after controlling for regional variability, LULC affects the occurrence of species within each foraging guild differently. For example, because gleaners pick food items from nearby substrate, they might respond to local habitat features not captured in our scale of investigation [44], and thus will not be affected by agricultural land cover *per se*. However because the elimination of vegetation on edges is common in intensified agriculture, we might find that they are negatively affected by high soybean proportions. Second, we predict that other guilds such as ground insectivores, omnivores, and aerial insectivores will be negatively affected by increasing agricultural land use. Third, we predict that exclusively granivorous species, such doves and pigeons, some even considered pests, benefit from agricultural land uses where food resources are available. Fourth, there is no agreement on previous studies on how raptors respond to agricultural land use, although they are very mobile, and many species have generalist diets so they could adapt to agricultural land uses. Finally, we assume that LULC effects on birds are constant over time, but we evaluate trends assuming that, if land use changes over the years of monitoring, avian occupancy will also be affected. However, if LULC is stable, changes in bird occupancy could be related to other variables independent to land use (e.g. population dynamics, agrochemicals).

This is the first long-term and large-scale study in Argentina evaluating the effects of agricultural land use on birds and their trends, incorporating imperfect detection probabilities using a community hierarchical occupancy approach. Our objective is to make inferences on potential ecosystem services provisioning—mainly seed dispersal (granivores), invertebrate and vertebrate pest control (insectivores and raptors), carcass and waste disposal (scavengers)–by evaluating the responses of species with similar ecological requirements. Ultimately, our results will provide valuable information for decision-making and implications for bird conservation in agricultural landscapes in central Argentina.

## Materials and Methods

### Study Area

The bird monitoring program in central Argentina (BMA) extends over sections of Entre Ríos, Santa Fe, Córdoba, La Pampa and Buenos Aires provinces between latitudes S37.375–30.783 and longitudes W64.819–58.267. Two ecoregions are represented in this area, the Pampas grassland, subdivided in Mesopotamic (northeast), Rolling (central), and Inland Pampas (western), and a small area of Espinal forest on the center of Santa Fe province, northeastern Córdoba and central Entre Ríos [45,46]. Pampas was originally dominated by grasses, and the Espinal by xerophytic forest with species like *Prosopis affinis*, *Acacia caven*, and *Geoffroea decorticans*; however, both regions have been highly modified by agricultural activities. The climate is mild, with a mean annual temperature of 10–20°C and a mean annual rainfall of around 1000 mm, which decreases to the south-west [12,25,46]. Initially, the BMA comprised 113,000 km^2^ in 2003–2004, increasing to 150,800 km^2^ in 2005, and to 255,000 km^2^ in 2006–2012 [21].

### Data Collection

**Fig 1:**
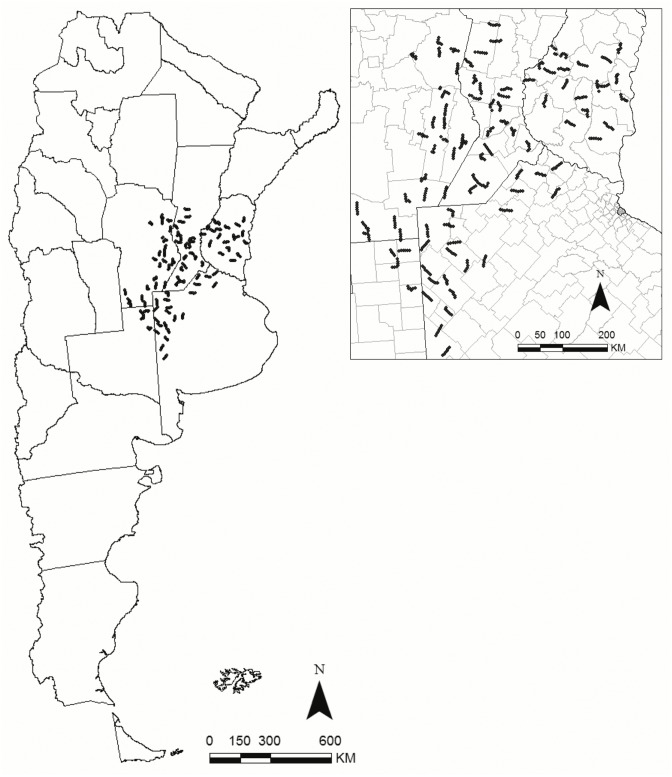
Regional bird monitoring program, indicating monitored routes, as of 2006–2012, covering an area 255,000 km^2^, over parts of Entre Ríos, Santa Fe, Córdoba, La Pampa and Buenos Aires provinces, Argentina.

In January each year from 2003 to 2012 (austral bird breeding season) we surveyed 47, 64 and 90 transects (2003–04, 2005, 2006–12, respectively) (Fig 1). Since transects were located along unpaved secondary and tertiary public roads, no specific permissions were required. Field studies did not involve endangered or protected species.

Transect locations were chosen applying a 30 km × 30 km grid over a map of the BMA area with previously defined strata consisting of agro-production zones and provincial boundaries. Grid cells were selected systematically every other cell. Within each selected cell, a 30 km route and the direction for the route to be surveyed were randomly selected [21]. Routes consisted of 30 points spaced every 1 km, where experienced surveyors recorded all bird species seen or heard, during 5 minutes. Surveys were conducted between 0600–1100 and 1500–2000, and every point was visited once.

LULC was recorded within a 200 m radius centered on each point, following bird sampling. We classified LULC in 7 categories: corn, soybean, and other annual crops (sunflower, wheat, sorghum, others), perennial pastures (alfalfa and alfalfa with other pasture legume), other pastures (natural grasslands, fallow), native forest, and planted forests (e.g. Eucalyptus). Other uses, such as aquatic, plowed fields or urban were excluded from the analyses because of their low representation in the region LULC recorded on the ground was validated using the LULC map developed with Landstat TM and ETM+ images for 2009 (Solari and Calamari *per*. *comm*.).

### Classification of avian groups and foraging guilds

We classified species into major groups with similar ecological requirements and taxonomy; then we identified guilds within groups according to their habitat preferences and foraging behavior. Each of the main groups were analyzed separately to increase computational efficiency, assuming that the parameter estimates for species may be more closely related to those of its group, considering the individual effects of species. We chose an all-species grouping within groups to avoid constraining parameters for species within a guild, focusing our interest on understanding species-level responses [47] allowing the identification of *a posteriori* responses common within and between guilds.

To build the main groups we separated Non Passeriformes from most Passeriformes (including a few species from Cuculidae, Picidae and Trochilidae families), and then separated species according to their main foraging resource (e.g. carnivores, granivores, insectivores) or substrate (e.g. ground, foliage). For example, we assumed there are substantial differences in the scale at which raptors or most doves and pigeons perceive the landscape compared to songbirds, especially foliage gleaners which pick food items from nearby substrates [43]. Six major groups were defined as follows: 1) raptors, 2) ground omnivores and herbivores, 3) ground granivores; 4) other granivores, 5) insectivores associated with foliage, 6) other insectivores. The first three are non-passerines and the last three were mostly passerines. Foraging guilds within those groups were identified by combining information on food preferences, strategy and main foraging stratum [43,48,49] (S1 Table).

### Statistical analysis

We used a hierarchical multi-species and multi-season occupancy model with a Bayesian approach to estimate the influence of LUCL on avian species over time [33–36]. In this context, hierarchical models are valuable because they improve inference by sharing information across species regardless of their relationships, which becomes especially important for those species less frequently detected in the community [32].

Occupancy estimation accounts for imperfect detection probabilities of each species (*p*<1), so that if a species is not observed at a certain point, it can be either truly absent, or present but undetected [29–31]. To achieve replication necessary to estimate detection probabilities at each location, we partitioned each 30 km route in segments (henceforth, ‘site’) of five points (*k* = 5). We chose five spatial replicates within each route because we anticipated that detection probabilities for many species would be low, resulting in imprecise estimates of occupancy for fewer replicates [31]. We understand that using spatial replicates could potentially violate the closure assumption (i.e., occupancy status of each site is constant across replicates) by confounding temporary absence with nondetection. However, there is disagreement regarding the extent to which spatial replication introduces bias in occupancy estimation [50–51]. In addition, the use of spatial replicates could introduce bias in the estimates if the replicates were spatially autocorrelated, but not when samples are independent, as we assume in this study given that the points were 1000 m apart to avoid double counting and dependence [52].

Site-occupancy (zero-inflated binomial) models can be formulated as a hierarchical state-space model, linking 2 binary regression models: a process model for occupancy of each species, and an observation model for detection conditional on occupancy [30,34]. Our process model assumes occupancy as a binary state *z(j*,*i*,*t)* for each species *i* = 1,2,…,*N* at site *j* = 1,2,…,*S* and year *t* = 1,2,…*Y*; where *z(j*,*i*,*t)* = 1 when the species is present and zero otherwise. This is a latent variable, since true occurrence it is imperfectly observed, modeled by a Bernoulli distribution with probability *ψ*_*j*,*i*,*t*_ that the species *i* occurs at site *j* and year *t*, specified as *z(j*,*i*,*t)* ~ Bern *(ψ*_*j*,*i*,*t*_ *)*. What we observe instead is *y(j*,*k*,*i*,*t)* at site *j*, point *k = 1*,*2*,*…*,*K*, for species *i* at year *t*. The observation model also follows a Bernoulli distribution as *y(j*,*k*,*i*,*t)* ~ Bern *(p*_*j*,*k*,*i*,*t*_. *z(j*,*i*,*t))*, with *p*_*j*,*k*,*i*,*t*_ as the probability that species *i* at site *j* is detected at point *k* on year *t*, and *y(j*,*k*,*i*,*t)* = 1 when the species is detected and zero otherwise. This formulation requires that the event of observing the species is conditional on the species being present (i.e., *z(j*,*i*,*t)* = 1).

We used a Bayesian approach in the programs R and JAGS, through package R2jags, which uses Markov chain Monte Carlo (MCMC) to find the posterior distribution of the parameters of interest [53]. We assumed that occurrence and detection probabilities for each species can be influenced by covariates, and modeled the effects using the logit-link function, where for example log *it*(*ψ*) = log(*ψ*/1−*ψ*)[35,36]. We summed LULC of all points *k* within each site *j* and divided by the total proportion of each site *j*. We discarded correlated covariates (Pearson *r* >0.5) leaving those that were more represented in the landscape, and modeled occupancy with the following predictors: proportion of soybean, corn, perennial pastures, and native forests. We dealt with the non-independence between the proportions of LULC (i.e., sum to one) by discarding three out of the seven categories, because they were represented in a low percentage as compared to the others. Similarly, we assumed that the probability of detecting a species was subject to forest coverage at each point, every year. We also incorporated latitude and longitude (centered on zero) to control for potential factors influencing occurrence of each species in addition to LULC. Because the monitoring program takes place in a large region, large scale drivers such as climate or original distribution of the species could affect occupancy [25,26]. For example, in the BMA area, mean temperatures in January are highly correlated to latitude (Pearson *r* = 0.977), while annual precipitation is correlated to longitude (Pearson *r* = 0.934; [54]). Our multi-season model assumed species occurrence to be conditional on temporal covariates only, allowing for differences in occupancy in different years and subject to land use changes [34,35]. We modified the model proposed by Kéry et al. [35] to incorporate random time effects on the baseline (i.e., intercept) occupancy and detection for each species on each site, as a means of controlling for potential sources of variation in different years (e.g. climate, observers). Our global occupancy model was:

$$\begin{array}{l} \text{logit}\psi \left(j,i,t\right)=u\left(i,t\right)+a1\left(i\right)*lat\left(j,t\right)+a2\left(i\right)*long\left(j,t\right)+a3\left(i\right)*soy\left(j,t\right)+a4\left(i\right)*corn\left(j,t\right)+\\ a5\left(i\right)*per\_past\left(j,t\right)+a6\left(i\right)*forest\left(j,t\right) \end{array}$$ (1)

where the parameters denoting covariates effects *a1* through *a6* for each species *i* = 1,2,…,*N* are estimated; *u* is estimated for each species *i* and year *t* = 1,2,…*Y* combination; location and LULC corresponds to each site *j* = 1,2,…,*S* and year *t*. We based our focus on estimation and prediction of the effects of covariates on each species, and in this context, we believe the best approach was to construct a global model and make the inferences based on 95% Bayesian credible intervals (95%BCI), since different species could respond differently to covariates, and average out their effect.

Similarly, we constructed the observation model as follows:

logitp(j,k,i,t)=v(i,t)+b1(i)*p_fors(j,k,t) (2)

where we estimated the effect of proportion of forest on detection *b1* for each species *i; v*, is estimated for each species *i* and year *t*; and the proportion of forest corresponds to each site *j*, year *t*, and point *k*.

The multi-species occupancy approach allows for the incorporation of a community hierarchical component in the model, where the species-level parameters are treated as random effects driven by covariates. Unlike most aforementioned applications of multi-species site occupancy models [33,35,36], we did not incorporate unobserved or rare species in the community, important for estimating species richness, not among our study objectives. We also discarded species associated with aquatic habitats, because our interest was on terrestrial birds; and those having fewer than 200 observations in the 10 year period, because inclusion of these sparsely-observed species created computational difficulties and added little to model performance. In addition, predicted occupancy and detection parameters for rare or hard-to-observe species tend towards the means of the parameters of the entire community adding no information about the effects of LULC to provide information for decision making.

In the analyses for each group, we assumed species parameter estimates could be more closely related between those species within a group, but still allow for individual species effects. For example, we assumed that *u(i*,*t)*~N*(μ*.*u (i)*, *σ*.*u(i))* followed a normal distribution where *μ*.*u (i)* and *σ*.*u(i)* are the mean and standard deviation across time, and similarly *μ*.*u (i)* ~N*(mu*.*μ*.*u*, *sigma*.*σ*.*u)* the hyper-parameters across the group of species.

We used independent flat (uninformative) priors for the group level hyper-parameters (S1 Appendix). We ran three chains of length 30,000 after a burn-in of 20,000, and thinned the posterior chains by 10 for economy of memory space and reduce autocorrelation to render 3000 iterations (9000 total for each parameter). We monitored convergence using the Gelman and Rubin diagnostic ($\hat{R}$), which includes the variance between the means from the parallel chains and the average of the within-chain variances, and convergence is reached when $\hat{R}$ is near 1 [55]. We also assessed model fit using a Bayesian p-value, which estimates the probability that the simulated data could be more extreme than the observed data [56].

## Results

We observed 263 species in the study area over a 10 year period, of which 205 are considered landbirds. We had sufficient data (>200 observations) to estimate occupancy for 74 landbird species (S1 Table).

**Fig 2:**
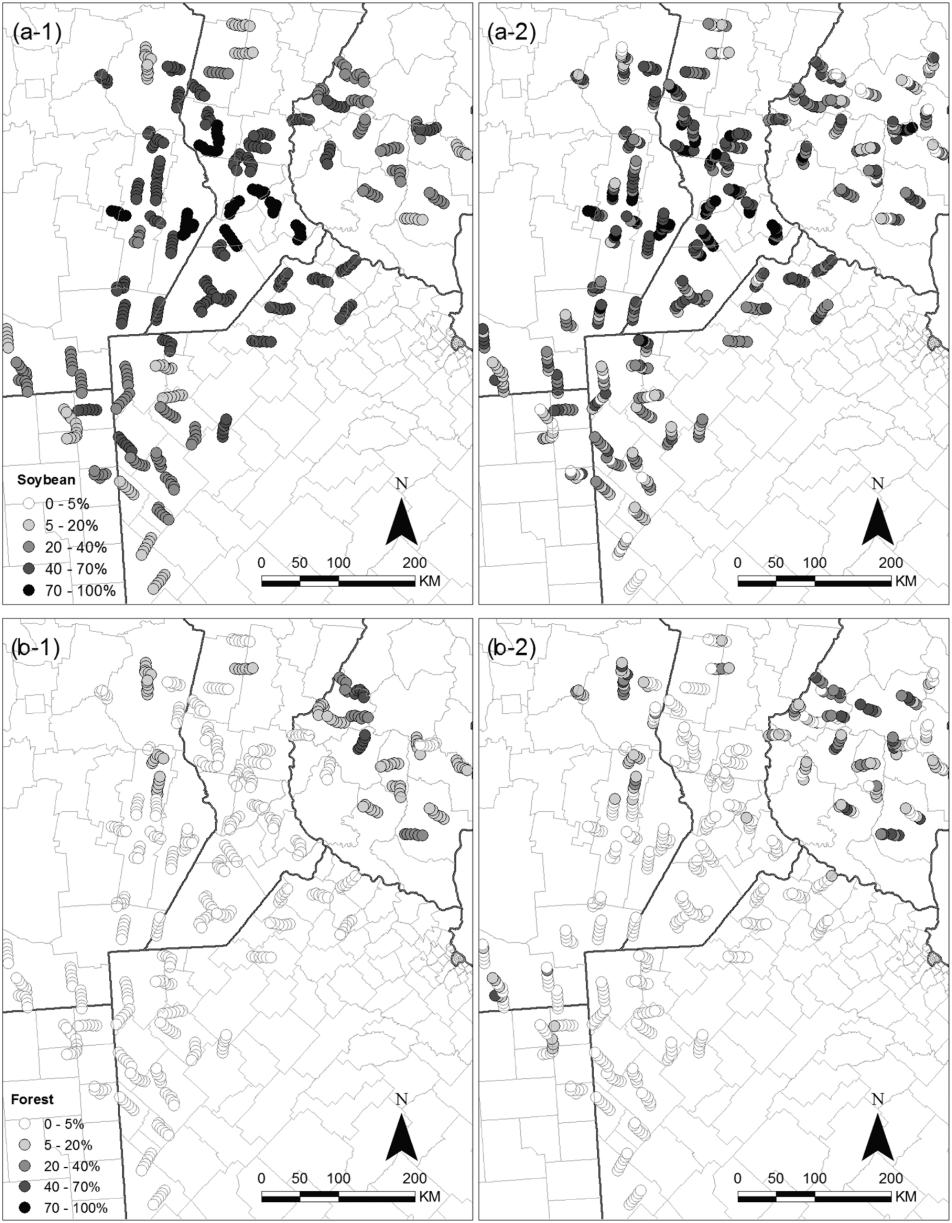
Land use and land cover proportion in the area of regional bird monitoring program in Pampas and Espinal ecoregions in Argentina. Land use of (a) soybean and land cover of (b) native forests for (1) 2006 and (2) 2012 is represented in percentage coverage in each site.

Soybean was distributed throughout the study area and to a larger degree in the central rolling Pampas region (Fig 2a). Native forests were more abundant in the Espinal area (Fig 2b). Perennial pastures were mostly in the north and to a lesser degree in the southwest; and corn had a patchy distribution throughout the region, which increased over time (S1 Fig). The proportion of soybean, corn, native forests, and perennial pastures recorded at sample points did not vary significantly throughout the 10 year period (S2 Fig). We did not include other annual crops (sunflower, wheat, sorghum, others), and other pastures (natural grasslands, fallow), in our analyses because these acreage were highly and negatively correlated to soybean. We also discarded planted forests (e.g. Eucalyptus) because we found inconsistencies among years and observers in the criteria by which percent cover was assigned by observers from different years, especially in the case of windbreaks.

### Detection probabilities

Mean detection probabilities were generally low, most < 0.5, and the response to percentage of forest cover varied by species (S2 Table and S3 Fig). The highest probabilities of detection were for the Eared Dove ~ 0.8, followed by Chimango Caracara, Southern Lapwing, and Rufous-collared Sparrow decreasing with forest cover. Detection probability for Picui Ground Dove, Monk Parakeet, and Rufous Hornero, as for several other species, increased rapidly with forest cover. This might be due to heterogeneity in species abundance, where the occurrence and detection probabilities of the species are likely to be correlated [33,36]. Incorporating time as a random effect on detection probabilities successfully absorbed variability in species detection brought about by factors not controlled by incorporating forest cover. For example, for unknown reasons, several species show a notably lower detection in 2007 (S4 and S5 Figs).

### Trends and regional bird occupancy

**Fig 3:**
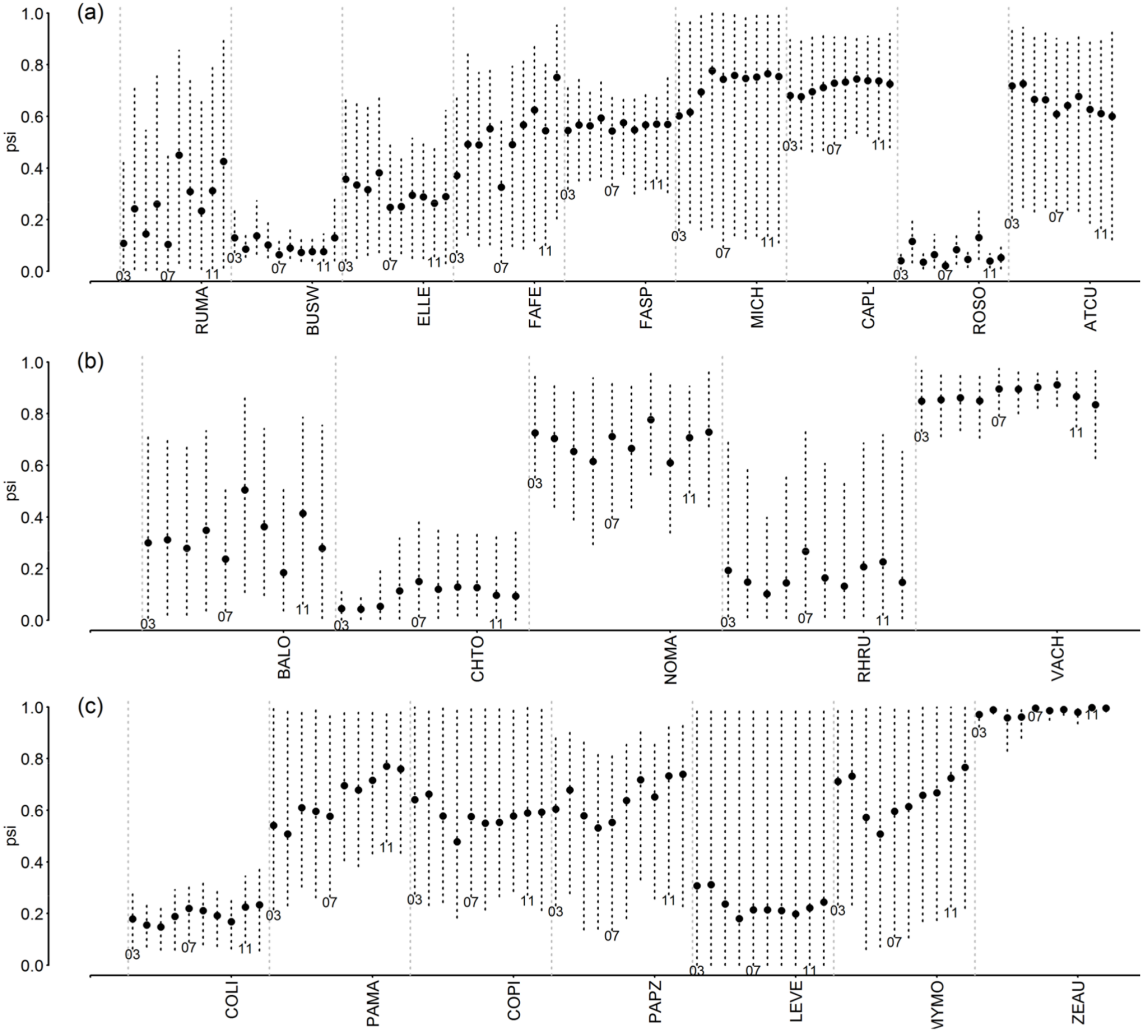
Mean occupancy ($\hat{\psi }$ ±SD, 95% BCI) for the complete study area during 2003–2012, in the regional bird monitoring program in Argentina for Non Passeriformes species. (a) Raptors; (b) ground omnivores and herbivores; (c) ground granivores. For details of species names and guilds, see S1 Table.

**Fig 4:**
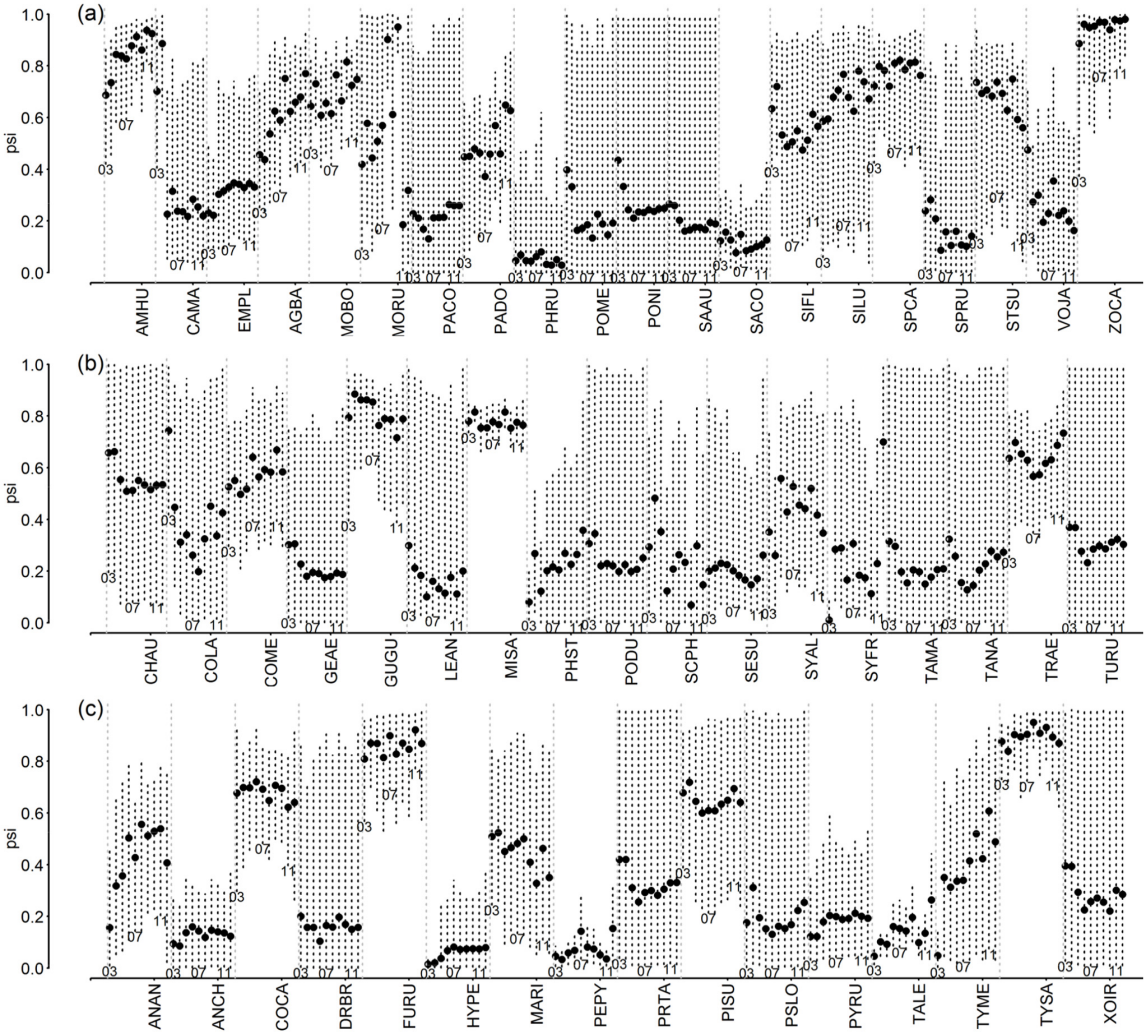
Mean occupancy ($\hat{\psi }$ ±SD, 95% BCI) for the complete study area during 2003–2012, in the regional bird monitoring program in Argentina for mostly Passeriformes species. (a) Granivores; (b) insectivores mostly associated with folliage; (c) other insectivores. For details of species names and guilds, see S1 Table.

We found no clear trends in mean occupancy response of species, or patterns among groups of species, in the study area for the period 2003–2012. Occupancy of most species remain constant, with a great amount of variability when sites were pooled from the larger area (Figs 3 and 4). Some species from different guilds such as Southern Lapwing, Eared Dove, Grassland and Rufous-collared Sparrows, Rufous Hornero, and Fork-tailed Flycatcher have high occupancy rates whereas other species, such as Swainson’s Hawk, Snail Kite, Southern Screamer, Rock Dove, White-tipped Plantcutter, Greyish Saltator, and insectivores such as Yellowish Pipit, Spectacled Tyrant, and Cliff Swallow, had low occupancy rates. Using years as random effect on the intercept of the occupancy models accounted for variation on occupancy that was not explained by LULC in the region (S6 and S7 Figs). For example, Spot-winged Pigeon, Monk Parakeet, Eared Dove, Grassland and House Sparrows, Bay-winged Cowbird, and Green-barred Woodpecker appear to be increasing over time whereas Dark-throated Seedeater, White-browed Blackbird, and Red-winged Tinamou, despite the variability, appear to be declining.

**Fig 5:**
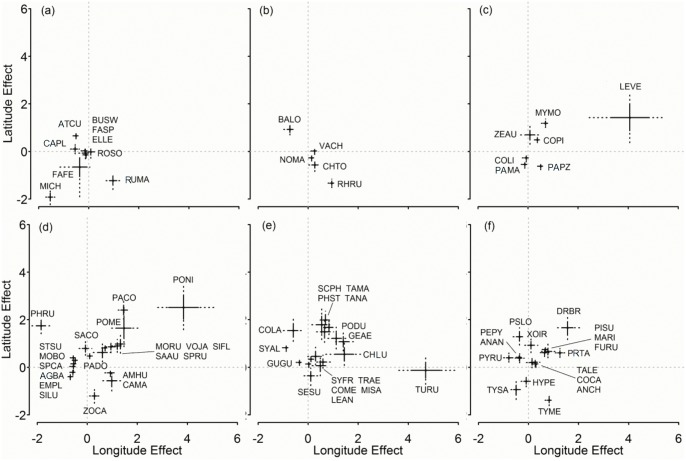
Latitude and longitude coefficients in the logit scale ($\hat{\beta }$ ±SD, 95% BCI) on logit occupancy (logit $\hat{\psi }$) of each bird species in the regional bird monitoring program in Argentina, 2003–2012. (a) Raptors; (b) ground omnivores and herbivores; (c) ground granivores; (d) other granivores; (e) insectivores mostly associated with folliage; (f) other insectivores. For details of species names and guilds, see S1 Table.

Latitude and longitude effects on occupancy reflect species occurrence along a geographic gradient (Fig 5). About half of the recorded raptor species (such as Chimango Caracara, Roadside Hawk, Burrowing owl, and Aplomado Falcon) occur infrequently in the northeast; whereas most granivore species, and insectivore gleaner species, Picui Ground Dove and White-tipped Dove, all associated with foliage, are common in that area. Other raptors such as Swainson’s Hawk, American Kestrel, White-tailed Kite and Snail Kite, and most ground omnivores exhibit weak or absent variation in occupancy over the ranges in latitude and longitude of this study. The Chimango Caracara, the Grassland Yellow Finch, a granivore, the Forked-tailed flycatcher, an aerial forager had higher occupancy probabilities in the southwest as compared to the northeast. The Burrowing owl, Upland Sandpiper, White-tipped Plantcutter and omnivores such as White-browed Blackbird, Dark-billed-cuckoo and Pale-breasted Spinetail are examples of species from different guilds with lower occupancy probabilities in the south and the east.

### Land use and land cover effects on occupancy

**Fig 6:**
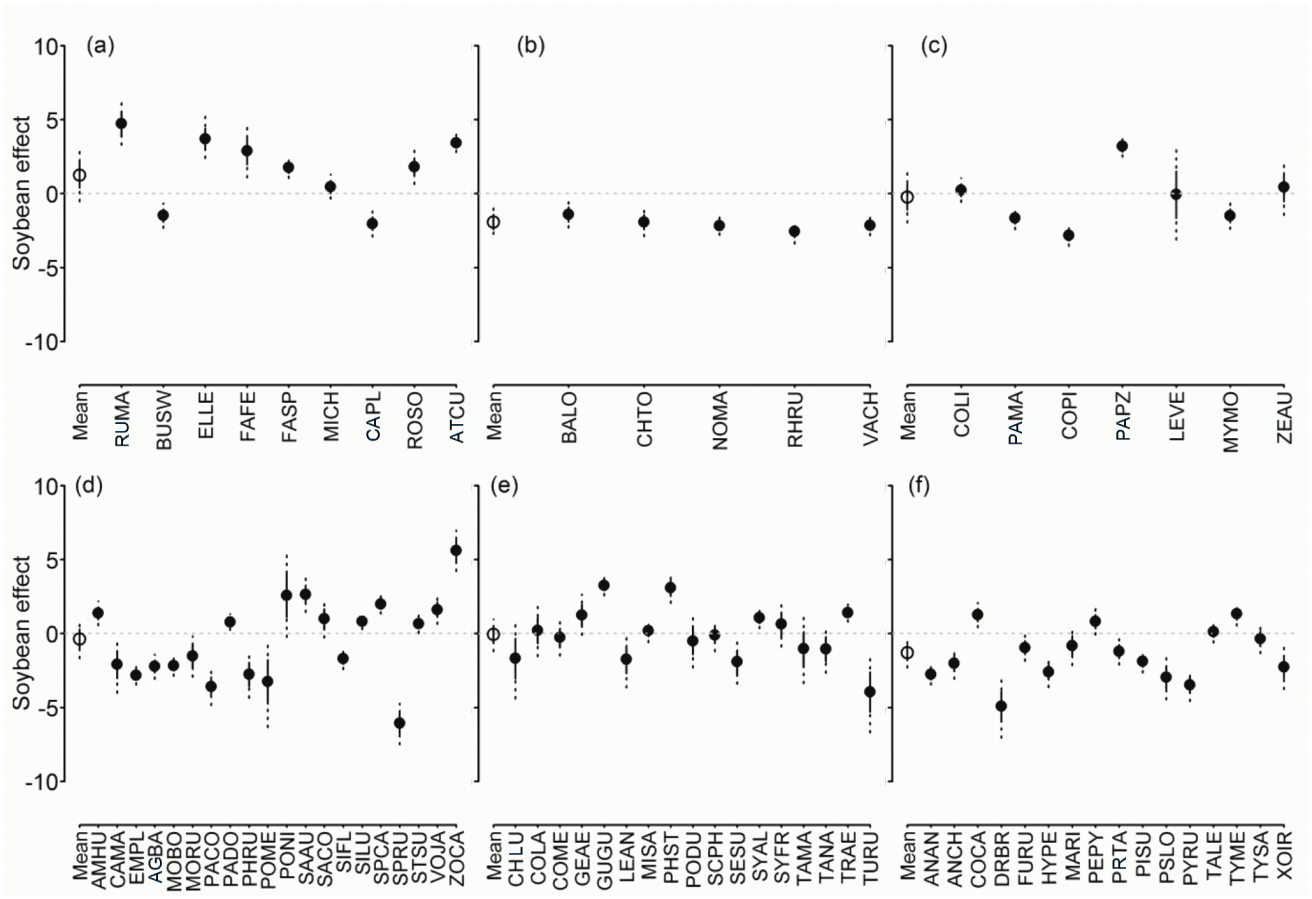
Soybean coefficients in the logit scale ($\hat{\beta }$ ±SD, 95% BCI) on logit occupancy (logit $\hat{\psi }$) of each bird species in the regional bird monitoring program in Argentina, 2003–2012. (a) Raptors; (b) ground omnivores and herbivores; (c) ground granivores; (d) other granivores; (e) insectivores mostly associated with folliage; (f) other insectivores. For details of species names and guilds, see S1 Table.

**Fig 7:**
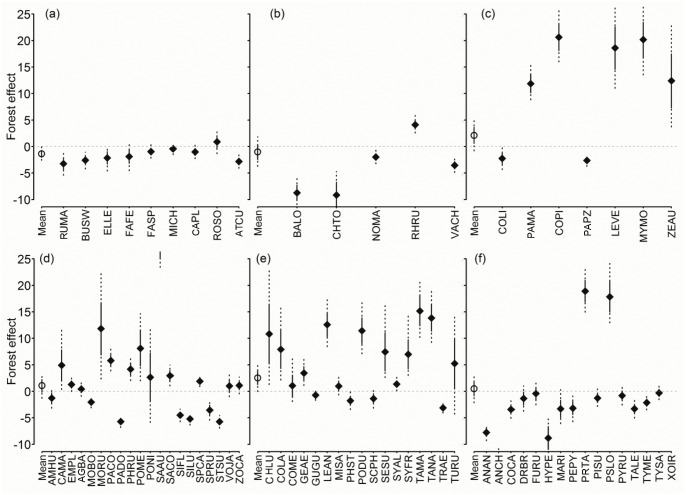
Native forest coefficients in the logit scale ($\hat{\beta }$ ±SD, 95% BCI) on logit occupancy (logit $\hat{\psi }$) of each bird species in the regional bird monitoring program in Argentina, 2003–2012. (a) Raptors; (b) ground omnivores and herbivores; (c) ground granivores; (d) other granivores; (e) insectivores mostly associated with folliage; (f) other insectivores. For details of species names and guilds, see S1 Table.

Generally, species responded more strongly to the effects of soybean and native forest coverage (Figs 6 and 7), and less to corn and perennial pastures (S8 and S9 Figs). Overall, 40% of landbirds were negatively affected by soybean, including all ground omnivores. Similarly, 30% appeared to be negatively affected by native forests, while 20% responded positively to soybean, including most raptors and some granivore gleaners; and 30% to native forests, half of which were insectivores. Only 20% of the species appeared to be negatively affected by corn whereas only three granivore gleaners, the Grassland Yellow Finch, Blue-black Grassquit, and Double-collared Seedeater appeared to be positively affected. Finally, 13% of all landbirds from different groups responded positively to perennial pastures and 8% negatively.

Only the Dark-throated Seedeater, a granivore gleaner, and the Spectacled Tyrant, an insectivorous sallier, were negatively affected by all agricultural uses examined (soybean, corn, perennial pastures). Similarly, species negatively affected by row crops included the Swainson’s Hawk, Southern Lapwing, Upland Sandpiper, and Spotted Nothura; the granivores Picui Ground Dove, Monk Parakeet, and Great Pampa Finch; and the insectivores Firewood-gatherer, Yellowish Pipit, and Great Kiskadee.

No raptor species seemed to benefit from native forests, and three species showed negative responses (Roadside and Swainson’s Hawks, and Burrowing Owl). Soybean had a negative effect on both the Swainson’s Hawk and the Southern Crested Caracara and corn on the Chimango Caracara. Two ground omnivore species showed positive responses to LULC, the Red-winged Tinamou to native forests, and the Spotted Nothura to perennial pastures. All Columbidae species, responded positively to native forests, except the Rock Dove which showed no change and the Picazuro Pigeon, the only species associated with soybean.

No generalizations could be made for granivore foliage gleaners’ responses. As a group, all insectivores associated with foliage showed a positive response to native forests most of which were and most consisted on foliage gleaners. In general, this group’s response to agricultural uses was highly variable. The rest of the insectivores generally responded negatively to soybean and corn percent area. Most ground insectivores and salliers were negatively affected by soybean, and some species by corn. Finally, aerial foragers responded weakly to agricultural uses, and only Brown-chested Martin was favored by native forest.

### Relevance to conservation decision making

**Fig 8:**
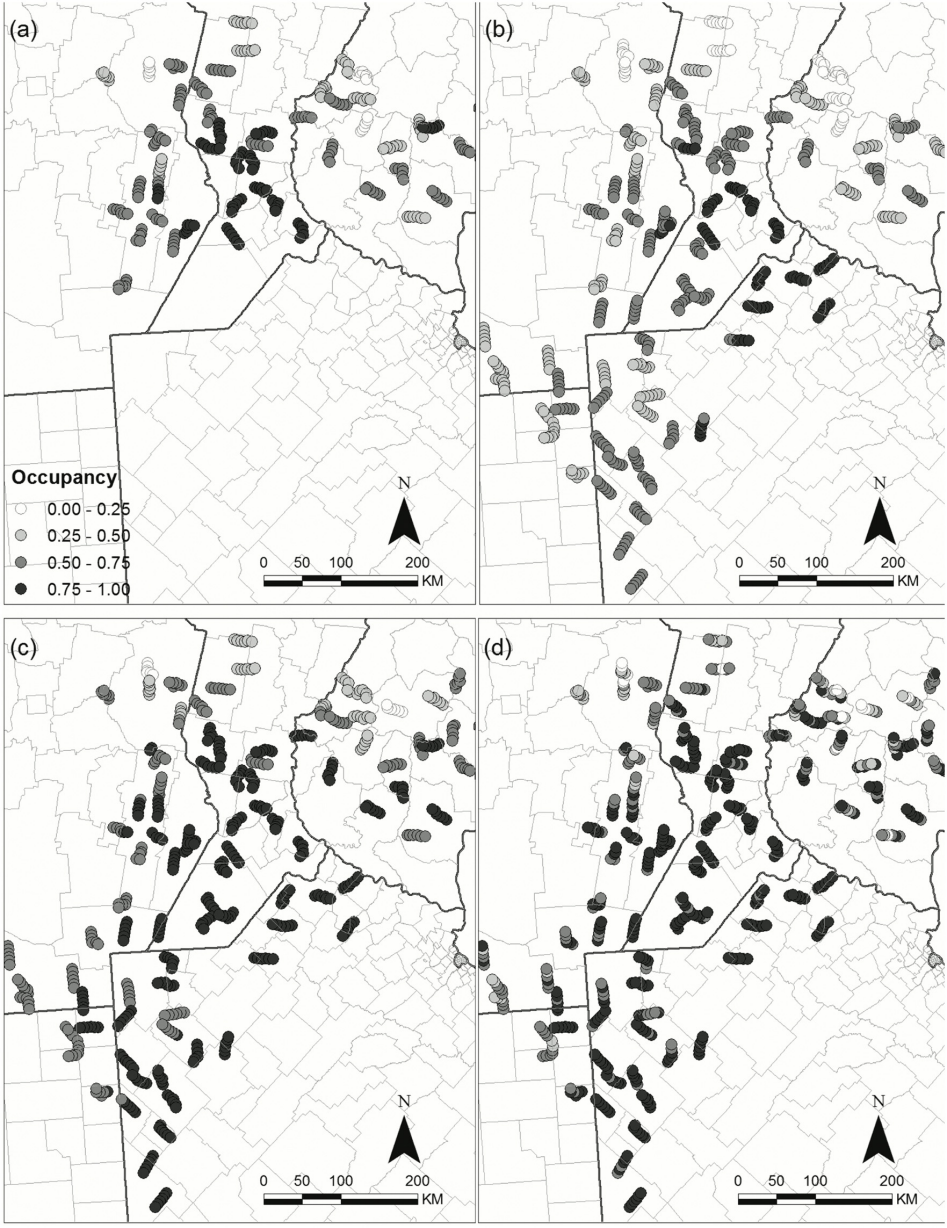
Picazuro Pigeon *Patagioenas picazuro* occupancy ($\hat{\psi }$) in the regional bird monitoring area in Argentina. Occupancy of the species is represented for (a) 2003, (b) 2006, (c) 2009, (d) 2012.

**Fig 9:**
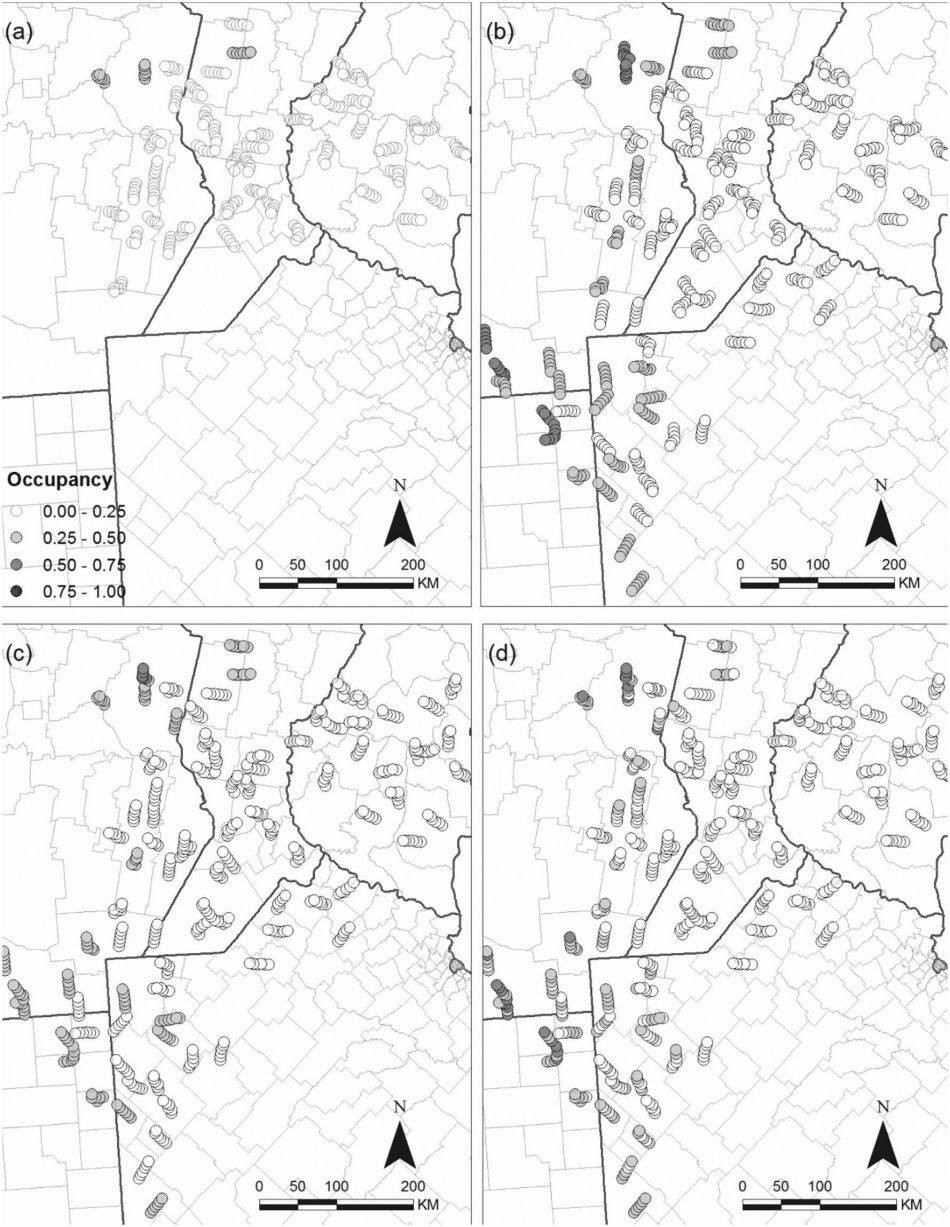
Vermilion Flycatcher *Pyrocephalus rubinus* occupancy ($\hat{\psi }$) in the regional bird monitoring area in Argentina. Occupancy of the species is represented for (a) 2003, (b) 2006, (c) 2009, (d) 2012.

In order to exemplify the potential application of our models to conservation, we illustrate the spatial response over time for a species of potential conservation concern (Vermilion flycatcher) and for a species of potential pest management concern (Picazuro pigeon) by evaluating their spatial distribution of occupancy probability throughout the region as it in relates to LULC. Picazuro pigeon is positively affected by soybean and negatively by native forests (Figs 6c and 7c); and the Vermilion flycatcher is negatively affected by soybean (Figs 6f and 7f). Occupancy probabilities of the Picazuro pigeon increase over most of the study area. However, they remain high in the central part where there is a higher proportion of soybean, and low in areas with natural forests (Fig 8). Conversely, the Vermilion flycatcher has a patchy distribution, with higher occupancy probabilities in the north and south west of the study area, where soybean proportions are lower. The response of this species over time does not appear to follow a clear trend, with some areas increasing and other decreasing in different years (Fig 9).

## Discussion

Our results support the hypothesis that birds are affected by the availability of food resources in agricultural landscapes in the Pampas and Espinal ecoregions of Argentina. Although many bird species within each guild responded differently to agricultural land use and cover, agreeing with previous studies of individual species [23–26, 28], we were able to identify some generalities for different trophic guilds. Most granivorous gleaners, passerines and non-passerine ground insectivores and omnivores responded negatively to high proportions of soybean, consistent with our prediction that loss of habitat heterogeneity results in lower site occupancy. On the other hand, many insectivore gleaners and aerial foragers seemed more tolerant to soy, and are presumably able to find suitable habitat or food within an intensified agricultural landscape. Exclusively granivorous species seemed to be affected differently at high proportions of soybean and corn, with the only positive response from Picazuro pigeon to soybean, while the rest were negatively affected, or unaffected. Last, raptors were favored by higher soybean proportions. The variability of individual species’ responses within groups, support the necessity to avoid *a priori* grouping of species into guilds [8,9]. By using current statistical approaches to model bird communities while accounting for imperfect detection [29,30], we were able to model individual responses, and use our data efficiently by linking species’ occupancy and detection probabilities in a hierarchical fashion. This allowed us to make inferences on species that we would have been unable to model with a single species approach [32,33,35,36]. We point out the importance of accounting for the imperfect detection of individual species. Making inferences assuming perfect detection could lead to bad management decisions regarding the conservation of a single species or a community assemblage [57].

Our study focuses attention on the conservation of species that appeared to be declining, had low occupancies, or were negatively affected by agricultural land use, in other words, those most sensitive to human related activities [58]. Trends could not be directly linked to agricultural land use or forest cover because the mean proportions were stable over time over BMA area, in spite of local changes. However, in the Rolling Pampas area, which has the longest agricultural history, our results agreed with those of previous studies and government statistics [11,12]. Some species with low occupancies in the region are habitat specialists such as Snail Kite or Southern Screamer, associated with wetlands; or White-tipped Plantcutter, and Greyish Saltator, associated with Espinal forests [59]. Some grassland species, such as Red-winged Tinamou, Dark-throated Seedeater, and White-browed Blackbird, although variable and not captured in broader analyses [28], seem to be declining. Others, such as Swainson’s Hawk, Yellowish Pipit, Spectacled Tyrant, and Cliff and White-rumped swallows had low occupancies, although widely distributed species—except Spectacled Tyrant—exhibited higher occupancy in the south. These results support previous studies documenting grassland species’ declines in southern South America [22], Europe [4,14], and North America [60]. All species were adversely affected particularly by soybean cultivation with the exception of swallows and White-browed Blackbird as indicated by Gavier-Pizarro et al. [26]. Dark-throated Seedeater and Spectacled Tyrant were affected by all agricultural uses including perennial pastures [28]. The Red-winged Tinamou is globally listed as of least concern but might be experiencing a population decline, in addition to over exploitation, and is associated to grasslands [24,58]. The Dark-throated Seedeater is considered to be a near threatened species, locally vulnerable, that is experiencing declines because of habitat loss and wild bird trade [58,61]. Finally Swainson’s Hawk is locally considered vulnerable as well and had suffered massive mortalities in the 1990s throughout the Pampas associated with insecticide poisoning via its foraging on grasshoppers and caterpillars [58,62]. The negative responses of these species to agricultural intensification, as represented by a greater proportion of soybean, and the overall low occupancy of some species in the agricultural landscape, support the hypothesis that some birds are adversely affected by intensification and the resulting loss of habitat heterogeneity, decrease in food resources for some species and increased agrochemical applications among other effects [1, 15, 16,40,63]. We suspect that the stable trends for some species, although potentially explainable by adaptations to agricultural landscapes, may be a consequence of the studied period which may have been too short to allow detection of trends, or due to a time lagged response to habitat transformation [3].

Species with high occupancy or increasing trends are tolerant to factors related to activity and are common in the Pampas and Espinal ecoregions [28,58,59]. Those with the highest occupancy resulted—and are often cited—as indifferent to agricultural intensification, or even associated to row crops. These included the Grassland and Rufous-collared Sparrows, the Rufous Hornero, the Eared Dove, and the Fork-tailed Flycatcher among others [22–24,26]. Species with increasing occupancy trends are granivores. They include the House Sparrow—an exotic species–, the Bay-winged Cowbird, the Eared Dove, and the Monk Parakeet. These results agree with studies in Europe stating that some species may even benefit from agriculture [14,64]. The Eared Dove and the Monk Parakeet are considered pests. As predicted, they showed an apparent increase in population growth and occupancy [65,66]. The Eared Dove does not appear to be adversely affected by crops of soybean or corn, but is associated with native forests. This agrees with previous studies that found it associated with agricultural lands with forest patches [25,26,67], but disagrees with studies that found a negative impact of agriculture [23]. The Monk Parakeet is found in the mesopotamic Pampas and Espinal ecoregions. Its presence is linked to the amount of native forests rather than the amount of corn and soybean, as stated in other studies [68]. Although this species is considered a pest for sunflower, corn, and fruits, among other crops, our surveys were conducted when the corn was in an early stage, which may explain its low occupancy in these areas [66]. With the exceptions of land use and cover proportions, which were stable for the whole area, increasing trends of both species could be linked to factors operating at a smaller scale such as the availability of small habitat patches or man-made structures, or large scale drivers, or even factors related to a higher breeding success of certain species as is often the case in exotic or pest species [65,68].

The remaining species exhibited different responses to agricultural land uses and native forest cover. A large number of species, including many granivore and insectivore foliage gleaners, were concentrated in the Espinal and mesopotamic Pampa ecoregions. These areas have a greater proportion of native forest remnants and intermediate levels of fragmentation, supporting the hypothesis relating increases in species diversity with increased habitat heterogeneity [12]. This also corresponds with a gradient of species richness from the wettest and hottest areas (northeast) to the driest and coolest areas (southwest) in this portion of Argentina [25]. Most raptors show a positive association to soybean as mentioned in other studies [25]. Generalist raptors could still find prey items [69], and those that roost in trees probably find isolated planted forest patches or windbreak trees not captured at our scale of analyses [44,70,71]. However, the roadside location of transects might have affected our results given there is a greater availability of perching sites (e.g. power lines; [70]) in those areas. Previous analyses found a negative but variable relationship between annual crops and raptors [27], which can be explained by the variability in the responses of different species. As predicted, ground omnivores and insectivores were affected by soybean and corn, most likely because their primary habitat (i.e., ground) is highly modified, affecting the availability of food and nesting grounds [72,73]. Despite being affected by row crops, species such as the Firewood-gatherer and Spotted Nothura are widely distributed, which could indicate that they find suitable habitats within the agricultural matrix not captured by our analyses, such as isolated trees for the first one, and other crops, natural grasslands or fallows for the latter [72]. On the other hand, most insectivore gleaners seemed unaffected by crops, contrary to our prediction. However this supports idea that those that perceive the landscape at finer scales could benefit from local spatial heterogeneity within agricultural landscapes [64], or persist in small patches of vegetation [74] and linear habitats such as field borders with natural vegetation. The latter might be more available in our roadside surveys [1,18,75].

We recognize that there are gaps in information regarding the real value of the ecosystem services provided by birds to agriculture in Argentina. However the diet composition of several species has been documented allowing us to infer their current consumption of pests, carrion and seed dispersal [76–79]. Some ecosystem services that could be provided by the subset of species we studied were seed dispersal, weed control, invertebrate and vertebrate pest control, carcass and waste disposal [6]. More than 80% of bird species are potential invertebrate pest controllers, followed in importance by many seed dispersors. These beneficial species were distributed throughout the study area. In addition, raptors may control rodent and other vertebrate pests despite agriculturization. However, almost half the insectivores are negatively affected by soybean, thus invertebrate pest control could be provided by few guilds and species in this region. For example, most ground insectivores, many salliers, and some granivore gleaners seemed to be absent from this crop. Conservation of avian diversity in agricultural landscapes is essential in order to maintain ecosystem functions and resilience; while guild diversity allows for exploitation of the resource in different ways (i.e., preying on different pests), diversity of species is important given the variability of responses associated with their particular traits [8]. To achieve a significant quantity on the service provided a numerical response is fundamental in order to control pest populations sufficiently; and this can be achieved either by either maintaining species richness or species abundance [6,80].

## Conclusions

The BMA program provides valuable information not only to evaluate temporal and regional effects of land transformation on birds, but also to provide methodologically rigorous evidence, including species-specific detection probabilities, to be incorporated into a decision making framework for conservation or management problems of species, or group of species, in agricultural landscapes in central Argentina. We illustrated how the temporal and spatial distribution of individual species can be mapped, to inform decision making or identify uncertainties to be explored in a future study. For example, by mapping the distribution of Vermilion flycatcher we were able to discover its patchy distribution, with higher occupancy probabilities in areas with low soybean proportions, but lower occurrence towards the Entre Ríos Espinal ecoregion. This suggests the preference of this species for open habitats but avoidance of soybean, and preference for small patches of woody vegetation or isolated trees in the agricultural matrix. This information is fundamental as a first step to elaborate a management plan for conservation.

Our results support the hypothesis that birds may be affected by the availability of food resources in agricultural landscapes in the Pampas and Espinal ecoregions of Argentina, given that we were able to find some generalities for different trophic groups. However, many species within the guilds responded differently to land use and native forest cover. Several species seem to have adapted or become tolerant to soybean and corn crops and perennial pastures; others have been negatively affected by agriculture, and others seem to respond on a larger or smaller scale. Some examples that emerge from our results and that could be incorporated into management plans are the cases of the Dark-throated Seedeater, the Red-winged Tinamou, and Swainson’s Hawk. The last of these species had low occupancy rates, and the first two appear to be declining; so that all three species give some degree of conservation concern [58]. Given the diversity of responses to land use and cover, maintaining habitat heterogeneity would likely benefit most species in an intensified agroecosystem. Heterogeneity at a landscape scale could be achieved with diversity of crops and pastures, while avoiding soybean monocultures, and natural areas within the agricultural matrix, native forests in Espinal ecoregion, and grasslands in Pampas. Conversely, species considered pests such as the Eared Dove and the Monk Parakeet show an apparent population growth and high occupancies, and should also be accounted for in management plans [65,66]. Management of these species is complex. There is a variety of actions that could be implemented, but there likely does not exist a single, practical solution that is both economically effective and offers immediate results; furthermore, success likely is both species-specific and scale dependent [81,82]. Information generated in our study could serve as a first step to identify the areas of greater concern and basic knowledge of species to delineate strategies for its future management.

Finally, we found that despite the fact that 80% of the species we studied are insectivores and thus potentially beneficial for pest control, a valuable ecosystem service in agricultural landscapes, some guilds such as ground insectivores are poorly represented. This fact evidences how agricultural intensification is affecting biodiversity and ecological functions, demanding management actions to recover it [83]. For example, we observed that many species are tolerant or benefit from agricultural uses such as perennial pastures; maintaining an heterogeneous agricultural landscape might help the conservation of sensitive groups. In addition, maintaining small patches of vegetation or vegetated borders would create habitat heterogeneity benefiting other groups that perceive the landscape on a smaller scale, such as gleaners, contributing to the conservation of bird diversity and its potential ecosystem services.

Although the effect of land use on birds in Pampa and Espinal ecoregions in Argentina has been studied in the past, this is the first long-term study of these characteristics, evaluating many species simultaneously [22]. In addition, although we now have ten years of data, we believe that the continuation of the BMA is essential to explore even longer trends that can capture potential time lags in the response to agriculturization by birds, or to assess the effects of the incorporation of new crop technologies, and confirm the predictive results of this study. With the information generated by BMA we will be able to continue to provide management solutions to populations and bird communities in agroecosystems as provided in the present study, and also identify key uncertainties to delineate future research opportunities. Given the dynamism of agroecosystems, it is essential to continue with the evaluation and monitoring of the effects of land use on birds, in order to anticipate the effects of changes in agricultural management in these landscapes in central of Argentina.

## Supporting Information

S1 AppendixR and JAGS model code and specifications, for our hierarchical Bayesian multi-species multi-season occupancy model to estimate the influence of LULC, over time, on avian species in Argentina, in the regional bird monitoring area, 2003–2012.We implemented the model using flat priors using program R2Jags. We ran three chains of the model for 30,000 iterations each after a burn-in of length 20,000 and thinned the model by 10. We assessed convergence of the model using $\hat{R}$.(DOCX)

S1 Database Proportion of forests used as a covariate to model detection probabilities of birds in the regional bird monitoring program in Argentina, 2003–2012. (CSV)

S2 Database Latitude and longitude (centered on zero), proportion of soybean, corn, perennial pastures, and native forests used as a covariate to model occupancy probabilities of birds in the regional bird monitoring program in Argentina, 2003–2012. (CSV)

S3 Database Data for raptor species used to model occupancy in the regional bird monitoring program in Argentina, 2003–2012. For details of species names and guilds, see S1 Table.
(CSV)

S4 Database Data for ground omnivore and herbivore species used to model occupancy in the regional bird monitoring program in Argentina, 2003–2012. For details of species names and guilds, see S1 Table.
(CSV)

S5 Database Data for ground granivore species used to model occupancy in the regional bird monitoring program in Argentina, 2003–2012. For details of species names and guilds, see S1 Table.
(CSV)

S6 Database Data for other granivore species used to model occupancy in the regional bird monitoring program in Argentina, 2003–2012. For details of species names and guilds, see S1 Table.
(CSV)

S7 Database Data for insectivore mostly associated with folliage species used to model occupancy in the regional bird monitoring program in Argentina, 2003–2012. For details of species names and guilds, see S1 Table.
(CSV)

S8 Database Data for other insectivore species used to model occupancy in the regional bird monitoring program in Argentina, 2003–2012. For details of species names and guilds, see S1 Table.
(CSV)

S1 Fig Land use proportion in the area of regional bird monitoring program in Pampas and Espinal ecoregions in Argentina. Land use of (a) corn and (b) perennial pastures for (1) 2006 and (2) 2012 is represented in percentage coverage in each site.
(TIF)

S2 Fig Proportion of land use and land cover (±SD) in 2003–2012, in the study area of the regional bird monitoring program in Argentina. Soybean (full triangle); corn (open circle); perennial pastures (full circle); native forests (open triangle).
(TIF)

S3 FigDetection probabilities ($\hat{p}$) as a function of proportion of forest cover in the regional bird monitoring program in Argentina, 2003–2012, for each bird species.(a) Raptors; (b) ground omnivores and herbivores; (c) ground granivores; (d) other granivores; (e) insectivores mostly associated with folliage; and (f) other insectivores. For details of species names and guilds, see S1 Table.(TIF)

S4 FigPosterior detection model intercepts ($\hat{p}$ ±SD, 95%BCI), in probability scale, incorporating time as a random effect, during 2003–2012, in the regional bird monitoring program in Argentina for Non Passeriformes species.(a) Raptors; (b) ground omnivores and herbivores; (c) ground granivores. For details of species names and guilds, see S1 Table.(TIF)

S5 FigPosterior detection model intercepts ($\hat{p}$ ±SD, 95%BCI), in probability scale, incorporating time as a random effect, during 2003–2012, in the regional bird monitoring program in Argentina for mostly Passeriformes species.(a) Granivores; (b) insectivores mostly associated with folliage; (c) other insectivores. For details of species names and guilds, see S1 Table.(TIF)

S6 FigPosterior occupancy model intercepts ($\hat{\psi }$ ±SD, 95% BCI), incorporating time as a random effect, during 2003–2012, in the regional bird monitoring program in Argentina for groups of Non Passeriformes species.(a) Raptors; (b) ground omnivores and herbivores; (c) ground granivores. For details of species names and guilds, see S1 Table.(TIF)

S7 FigPosterior occupancy model intercepts ($\hat{\psi }$ ±SD, 95% BCI), incorporating time as a random effect, during 2003–2012, in the regional bird monitoring program in Argentina for mostly Passeriformes species.(a) Granivores; (b) insectivores mostly associated with folliage; (c) other insectivores. For details of species names and guilds, see S1 Table.(TIF)

S8 FigCorn coefficients in the logit scale ($\hat{\beta }$ ±SD, 95% BCI) on logit occupancy (logit $\hat{\psi }$) of each bird species in the regional bird monitoring program in Argentina, 2003–2012.(a) Raptors; (b) ground omnivores and herbivores; (c) ground granivores; (d) other granivores; (e) insectivores mostly associated with folliage; (f) other insectivores. For details of species names and guilds, see S1 Table.(TIF)

S9 FigPerennial pastures coefficients in the logit scale ($\hat{\beta }$ ±SD, 95% BCI) on logit occupancy (logit $\hat{\psi }$) of each bird species in the regional bird monitoring program in Argentina, 2003–2012.(a) Raptors; (b) ground omnivores and herbivores; (c) ground granivores; (d) other granivores; (e) insectivores mostly associated with folliage; (f) other insectivores. For details of species names and guilds, see S1 Table.(TIF)

S1 Table Avian species observed in the regional monitoring program in Pampas and Espinal regions, in Argentina from 2003–2012 (registered >200 times). Groups for analyses purposes and guilds are indicated: raptors (RAP), ground omnivores and herbivores (OMN), ground granivores (GRA2), other granivores (GRA), insectivores mostly associated with folliage (INS1) and other insectivores (INS2) [43,48,49].
(DOCX)

S2 TablePosterior proportion of forest effects on detection probabilities in the logit scale (logit($\hat{\beta }$_*forest*_)), with upper and lower 95% BCI (LBCI, UBCI) for each species in the regional bird monitoring program in Argentina, 2003–2012.(DOCX)
