# Supplementary material for: Multi-Season Regional Analysis of Multi-Species Occupancy: Implications for Bird Conservation in Agricultural Lands in East-Central Argentina
Source: PLoS One. 2015 Jun 18;10(6):e0130874. doi: 10.1371/journal.pone.0130874 (PMC4472512; doi:10.1371/journal.pone.0130874)
Supplement: S1 Appendix — We implemented the model using flat priors using program R2Jags. We ran three chains of the model for 30,000 iterations each after a burn-in of length 20,000 and thinned the model by 10. We assessed convergence of the model using R^. (DOCX) [file pone.0130874.s001.docx]

sink("global.jags")

cat("

#JAGS code starts

model

{

## Model missing covariates (some examples as an illustration)

for (j in 1:6) {

for (k in 1:5) {

pfor[j,k,1]~dunif(0,1)

}}

#...

for (j in 1:6){

soy[j,1]~dunif(0,0.5)

}

#…

for (j in 1:6){

corn[j,1]~dunif(0,0.5)

}

#…

for (j in 314:318){

forest[j,8]~dunif(0,0.5)

}

#…

for (j in 1:6){

per_past[j,1]~dunif(0,0.5)

}

#…

## Prior distributions

mean.mu.u~ dunif(0, 1)

mean.u <- log(mean.mu.u)- log(1-mean.mu.u)

mean.mu.v ~ dunif(0, 1)

mean.v <- log(mean.mu.v)- log(1-mean.mu.v)

mu.a1 ~ dnorm(0, 0.37)

mu.a2 ~ dnorm(0, 0.37)

mu.a3 ~ dnorm(0, 0.37)

mu.a4 ~ dnorm(0, 0.37)

mu.a5 ~ dnorm(0, 0.37)

mu.a6 ~ dnorm(0, 0.37)

mu.b1 ~ dnorm(0, 0.37)

sd.a1~dunif(0,10)

tau.a1<-pow(sd.a1,-2)

sd.a2~dunif(0,10)

tau.a2<-pow(sd.a2,-2)

sd.a3~dunif(0,10)

tau.a3<-pow(sd.a3,-2)

sd.a4~dunif(0,10)

tau.a4<-pow(sd.a4,-2)

sd.a5~dunif(0,10)

tau.a5<-pow(sd.a5,-2)

sd.a6~dunif(0,10)

tau.a6<-pow(sd.a6,-2)

sd.b1~dunif(0,10)

tau.b1<-pow(sd.b1,-2)

sd.mu.u~dunif(0,10)

tau.mu.u<-pow(sd.mu.u,-2)

sd.mu.v~dunif(0,10)

tau.mu.v<-pow(sd.mu.v,-2)

for (i in 1:n){

mu.u[i] ~ dnorm(mean.u, tau.mu.u)

mu.v[i] ~ dnorm(mean.v, tau.mu.v)

a1[i] ~ dnorm (mu.a1, tau.a1)

a2[i] ~ dnorm (mu.a2, tau.a2)

a3[i] ~ dnorm (mu.a3, tau.a3)

a4[i] ~ dnorm (mu.a4, tau.a4)

a5[i] ~ dnorm (mu.a5, tau.a5)

a6[i] ~ dnorm (mu.a6, tau.a6)

b1[i]~dnorm(mu.b1,tau.b1)

sd.u[i]~dunif(0,10)

sd.v[i]~dunif(0,10)

tau.u[i]<-pow(sd.u[i],-2)

tau.v[i]<-pow(sd.v[i],-2)

for (t in 1:Y){

u[i,t] ~ dnorm(mu.u[i], tau.u[i])

v[i,t] ~ dnorm(mu.v[i], tau.v[i])

}

## Process model

for (j in 1:site) {

for (t in 1:Y){

z[j,i,t]~dbern(psi[j,i,t])

logit(psi[j,i,t]) <- u[i,t] + a1[i]*lat[j,t]+ a2[i]*long[j,t]+ a3[i]*soy[j,t]+ a4[i]*corn[j,t]+ a5[i]*per_past[j,t]+ a6[i]*forest[j,t]

## Observation model

for (k in 1:R) {

y[j,k,i,t] ~ dbern(mu.y[j,k,i,t])

mu.y[j,k,i,t] <- p[j,k,i,t]*z[j,i,t]

logit(p[j,k,i,t]) <- v[i,t] + b1[i]*pfor[j,k,t]

## Observed deviance

dev[j,k,i,t]<-y[j,k,i,t]*log(mu.y[j,k,i,t])+(1-y[j,k,i,t])*log(1-mu.y[j,k,i,t])

### Predict new observation and compute deviance

y.new[j,k,i,t] ~ dbern(mu.y[j,k,i,t])

dev.sim[j,k,i,t]<- y.new[j,k,i,t]*log(mu.y[j,k,i,t])+(1-y.new[j,k,i,t])*log(1-mu.y[j,k,i,t])

} #R

}#year

}#site

}#species

sum.dev<-sum(dev[,,,])

sum.dev.sim<-sum(dev.sim[,,,])

test<-step(sum.dev.sim - sum.dev)

bpvalue<-mean(test)

} #model

",fill=TRUE)

sink()

## Create the necessary arguments to run the jags() command in R

### Load data

sp.data = list(y=y, R=R, site=site , n=n ,Y=Y, pfor=pfor, lat=lat, long=long, soy=soy, corn=corn, per_past=per_past, forest=forest)

## Initialize z to be sites where at least 1 detection

zst<-array(0,dim=c(site,n,Y))

y<-y

for (i in 1:site) {

for (s in 1:n) {

for (t in 1:Y) {

zst[i,s,t]<-(sum(y[i,,s,t])>0)*1

}}}

zst[is.na(zst)]<-1

## Specify the initial values for the chains

inits1<- list(z=zst,mean.mu.u=runif(1,0,1),mean.mu.v=runif(1,0,1),mu.a1=runif(1,-2,2),mu.a2=runif(1,-2,2),mu.a3=runif(1,-2,2), mu.a4=runif(1,-2,2), mu.a5=runif(1,-2,2),mu.a6=runif(1,-2,2),mu.b1=runif(1,-2,2),sd.mu.u=runif(1,0.1,5),sd.mu.v=runif(1,0.1,5),

sd.a1=runif(1,0.1,5),sd.a2=runif(1,0.1,5),sd.a3=runif(1,0.1,5),sd.a4=runif(1,0.1,5),

sd.a5=runif(1,0.1,5),sd.a6=runif(1,0.1,5),sd.b1=runif(1,0.1,5),sd.u=runif(n,0.1,5),sd.v=runif(n,0.1,5))

inits2<- list(z=zst,mean.mu.u=runif(1,0,1),mean.mu.v=runif(1,0,1),mu.a1=runif(1,-2,2),mu.a2=runif(1,-2,2),mu.a3=runif(1,-2,2), mu.a4=runif(1,-2,2),mu.a5=runif(1,-2,2), mu.a6=runif(1,-2,2),mu.b1=runif(1,-2,2),sd.mu.u=runif(1,0.1,5),sd.mu.v=runif(1,0.1,5), sd.a1=runif(1,0.1,5), sd.a2=runif(1,0.1,5), sd.a3=runif(1,0.1,5),sd.a4=runif(1,0.1,5), sd.a5=runif(1,0.1,5),sd.a6=runif(1,0.1,5), sd.b1=runif(1,0.1,5), sd.u=runif(n,0.1,5), sd.v=runif(n,0.1,5))

inits3<- list(z=zst,mean.mu.u=runif(1,0,1),mean.mu.v=runif(1,0,1),mu.a1=runif(1,-2,2),mu.a2=runif(1,-2,2),mu.a3=runif(1,-2,2), mu.a4=runif(1,-2,2),mu.a5=runif(1,-2,2), mu.a6=runif(1,-2,2),mu.b1=runif(1,-2,2),sd.mu.u=runif(1,0.1,5),sd.mu.v=runif(1,0.1,5),

sd.a1=runif(1,0.1,5),sd.a2=runif(1,0.1,5),sd.a3=runif(1,0.1,5),sd.a4=runif(1,0.1,5),

sd.a5=runif(1,0.1,5),sd.a6=runif(1,0.1,5),sd.b1=runif(1,0.1,5),sd.u=runif(n,0.1,5),sd.v=runif(n,0.1,5))

inits<-list(inits1,inits2,inits3)

## Specify the parameters to be monitored

sp.params <- c("mean.mu.u","mean.mu.v", "mu.a1", "mu.a2", "mu.a3", "mu.a4", "mu.a5", "mu.a6","mu.b1", "sd.mu.u", "sd.u", "sd.v","sd.a1","sd.a2", "sd.a3","sd.a4", "sd.a5", "sd.a6", "sd.b1","u","v","a1","a2","a3","a4","a5","a6","b1","test","bpvalue")

##Run the model and call the results “fit”

fit = jags(sp.data, inits, sp.params, "global.jags", n.chains=3, n.iter=50000, n.burnin=30000, n.thin=10)
