# Supplementary figures and images for: Multi-Season Regional Analysis of Multi-Species Occupancy: Implications for Bird Conservation in Agricultural Lands in East-Central Argentina
Source: PLoS One. 2015 Jun 18;10(6):e0130874. doi: 10.1371/journal.pone.0130874 (PMC4472512; doi:10.1371/journal.pone.0130874)

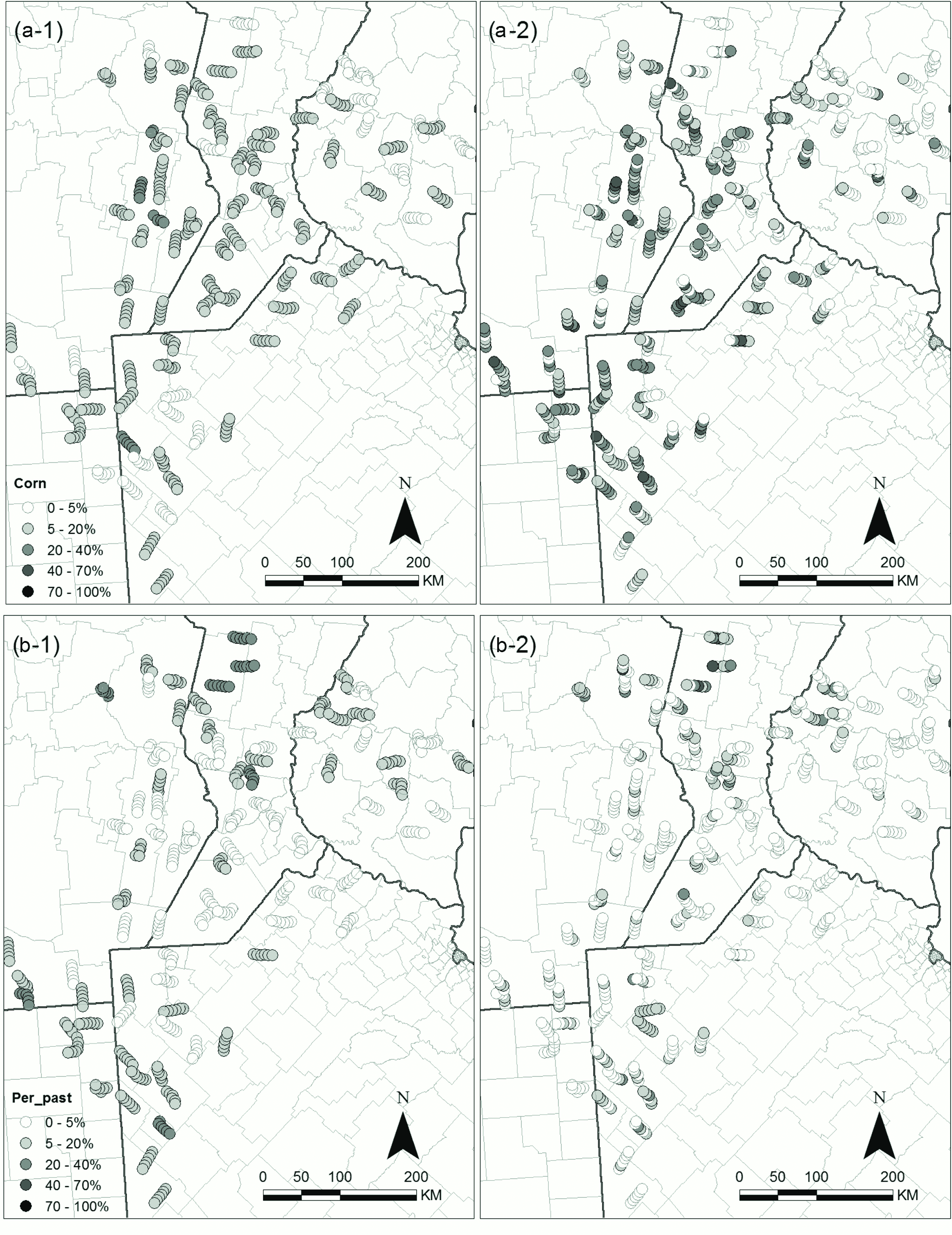

Supplement: S1 Fig — Land use of (a) corn and (b) perennial pastures for (1) 2006 and (2) 2012 is represented in percentage coverage in each site. (TIF) [file pone.0130874.s010.tif]

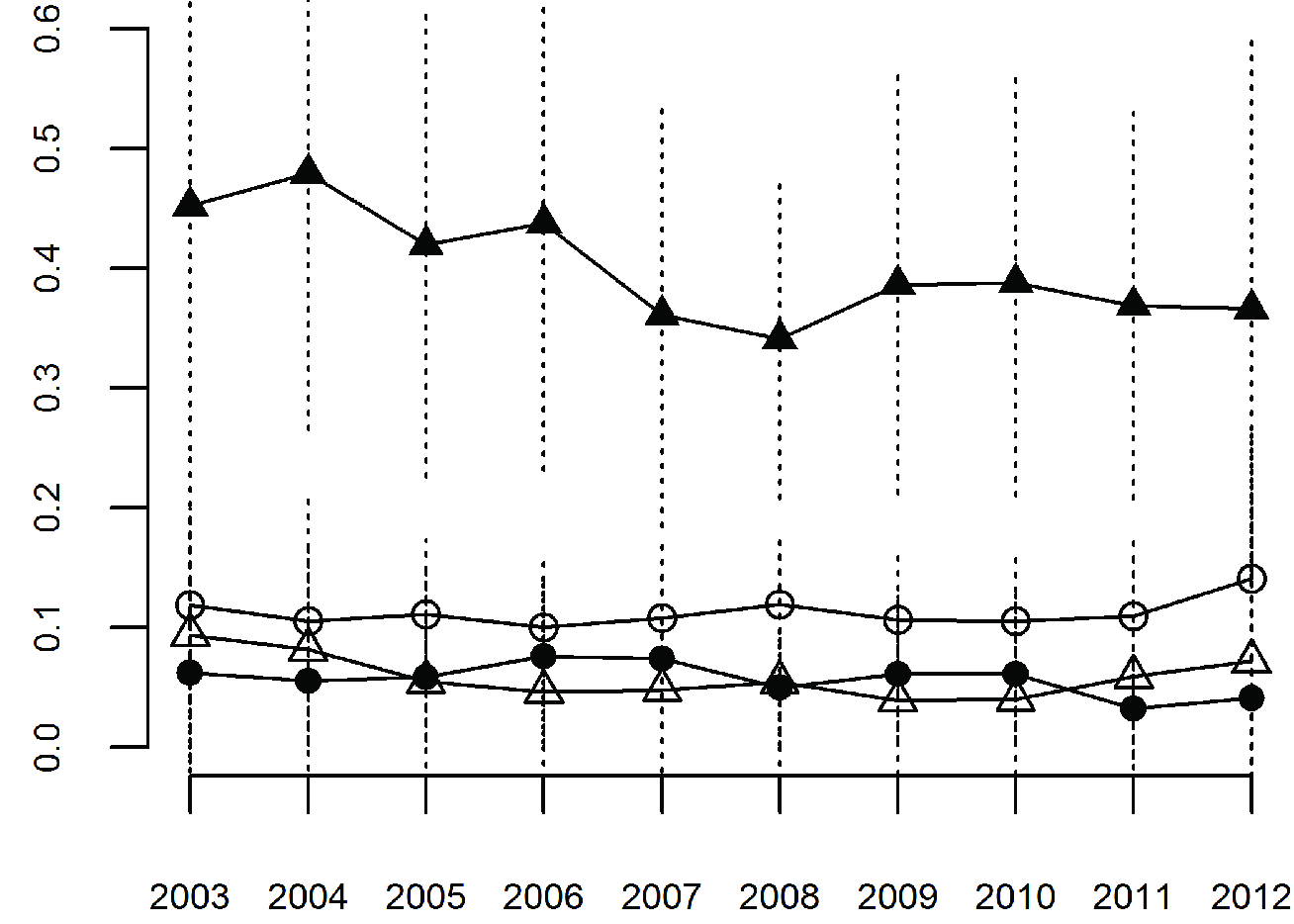

Supplement: S2 Fig — Soybean (full triangle); corn (open circle); perennial pastures (full circle); native forests (open triangle). (TIF) [file pone.0130874.s011.tif]

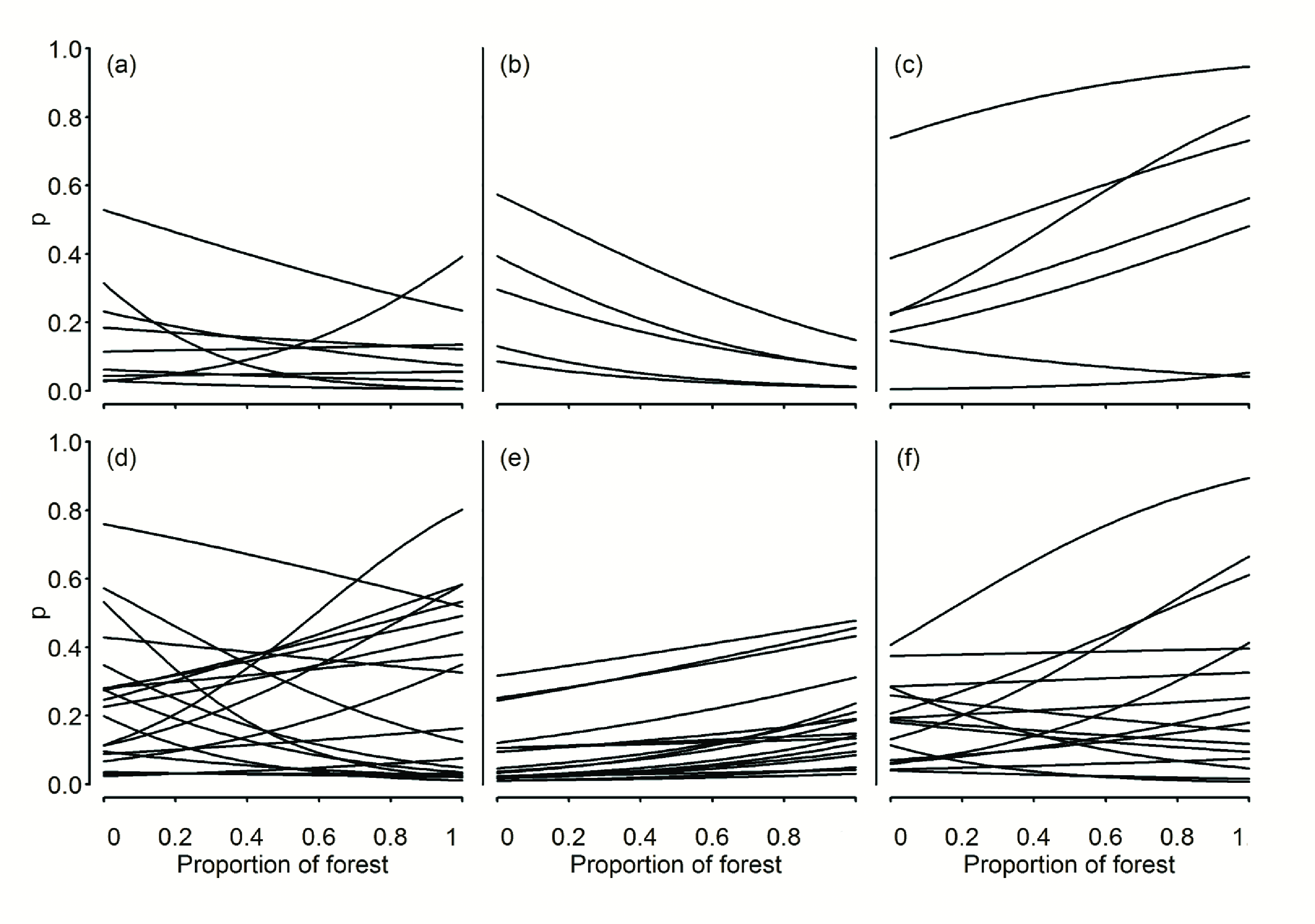

Supplement: S3 Fig — (a) Raptors; (b) ground omnivores and herbivores; (c) ground granivores; (d) other granivores; (e) insectivores mostly associated with folliage; and (f) other insectivores. For details of species names and guilds, see S1 Table. (TIF) [file pone.0130874.s012.tif]

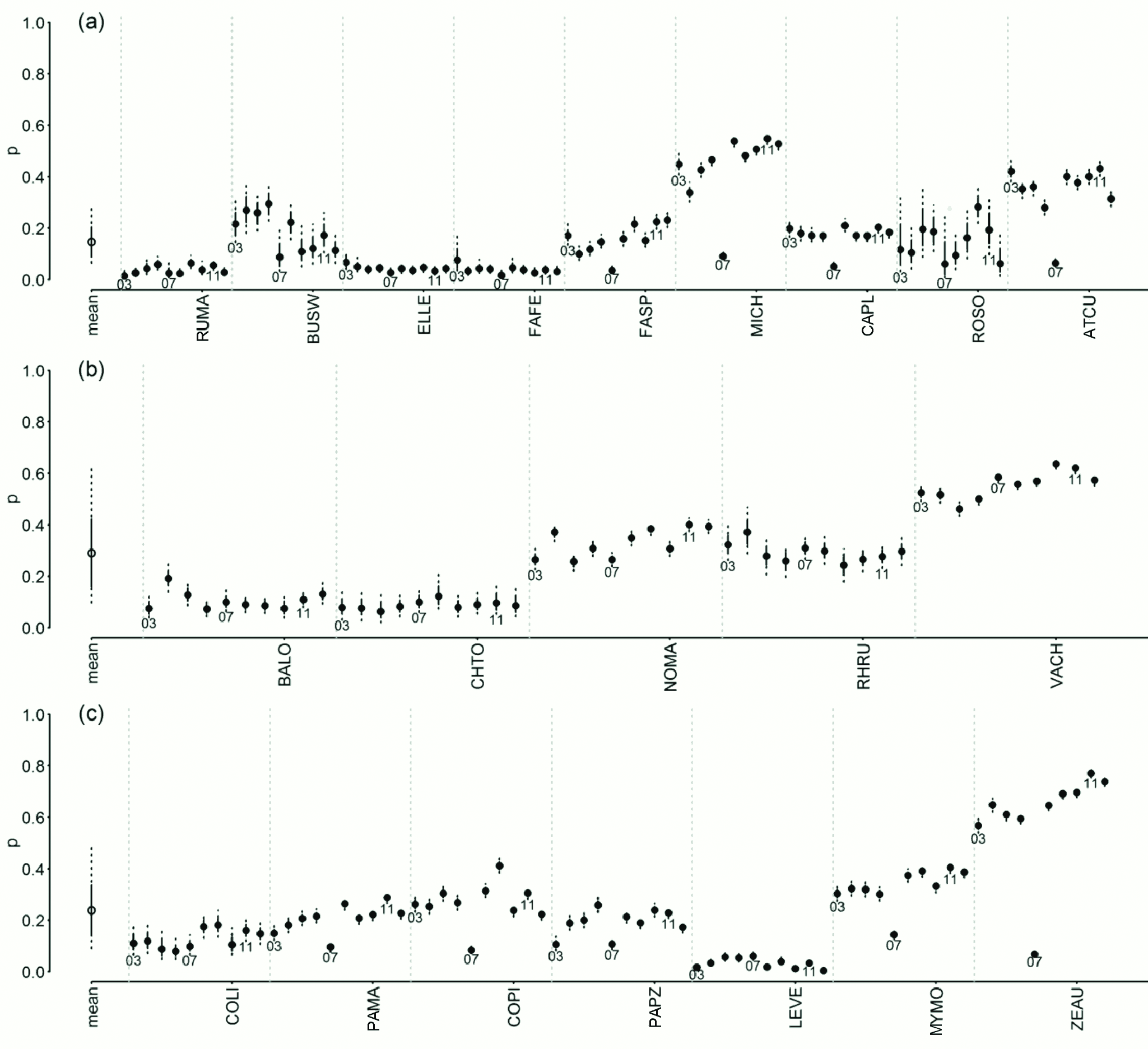

Supplement: S4 Fig — (a) Raptors; (b) ground omnivores and herbivores; (c) ground granivores. For details of species names and guilds, see S1 Table. (TIF) [file pone.0130874.s013.tif]

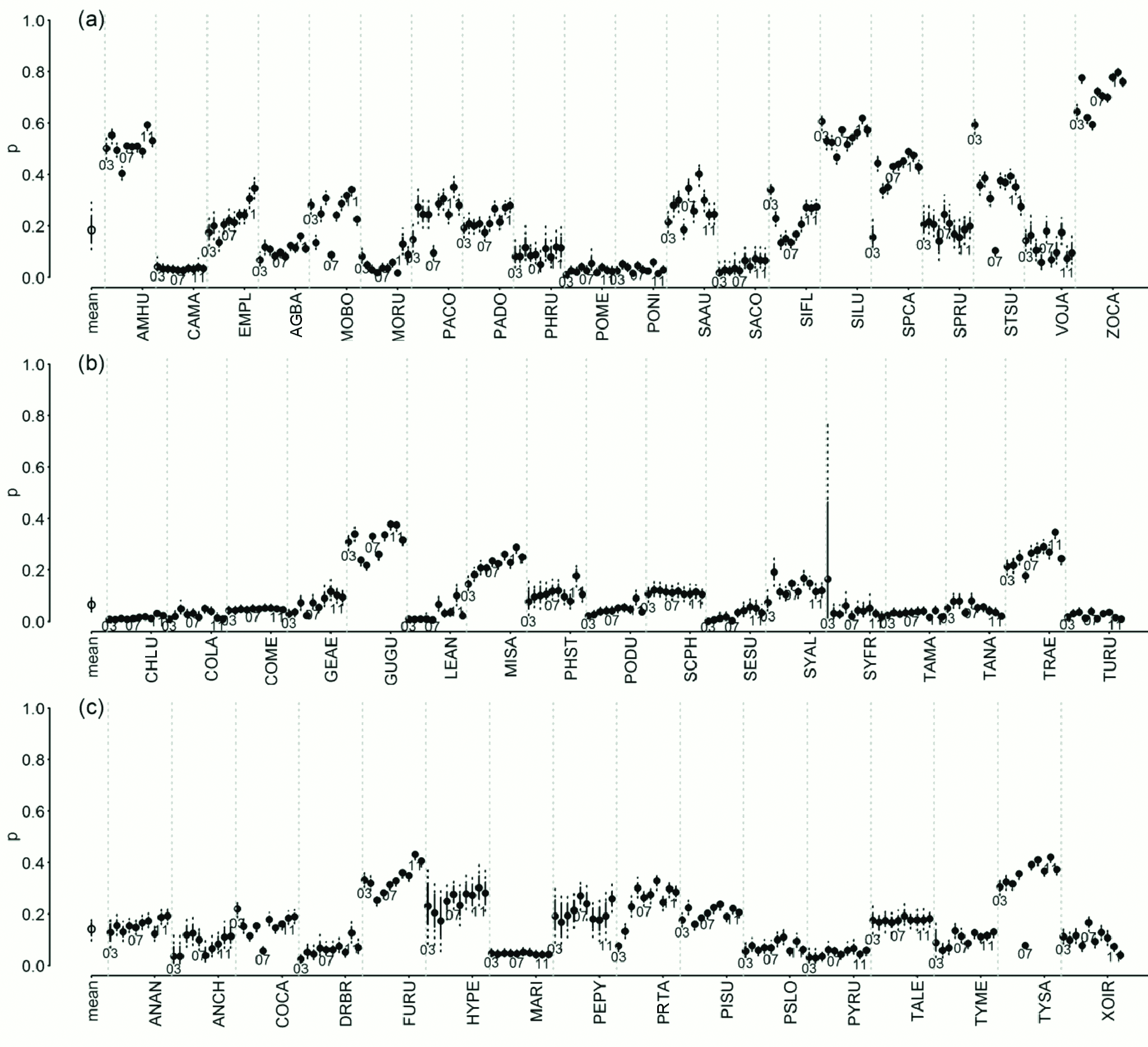

Supplement: S5 Fig — (a) Granivores; (b) insectivores mostly associated with folliage; (c) other insectivores. For details of species names and guilds, see S1 Table. (TIF) [file pone.0130874.s014.tif]

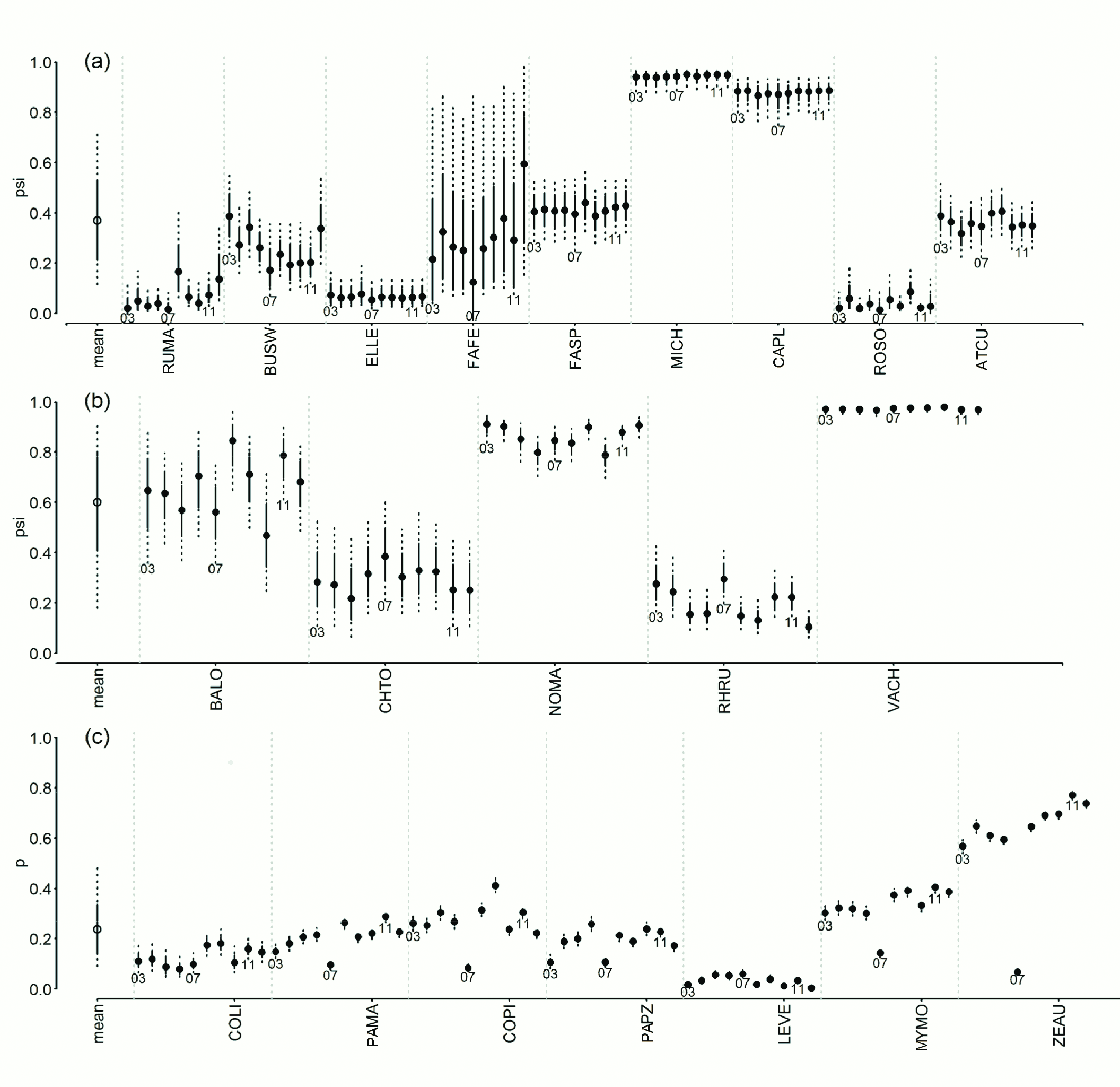

Supplement: S6 Fig — (a) Raptors; (b) ground omnivores and herbivores; (c) ground granivores. For details of species names and guilds, see S1 Table. (TIF) [file pone.0130874.s015.tif]

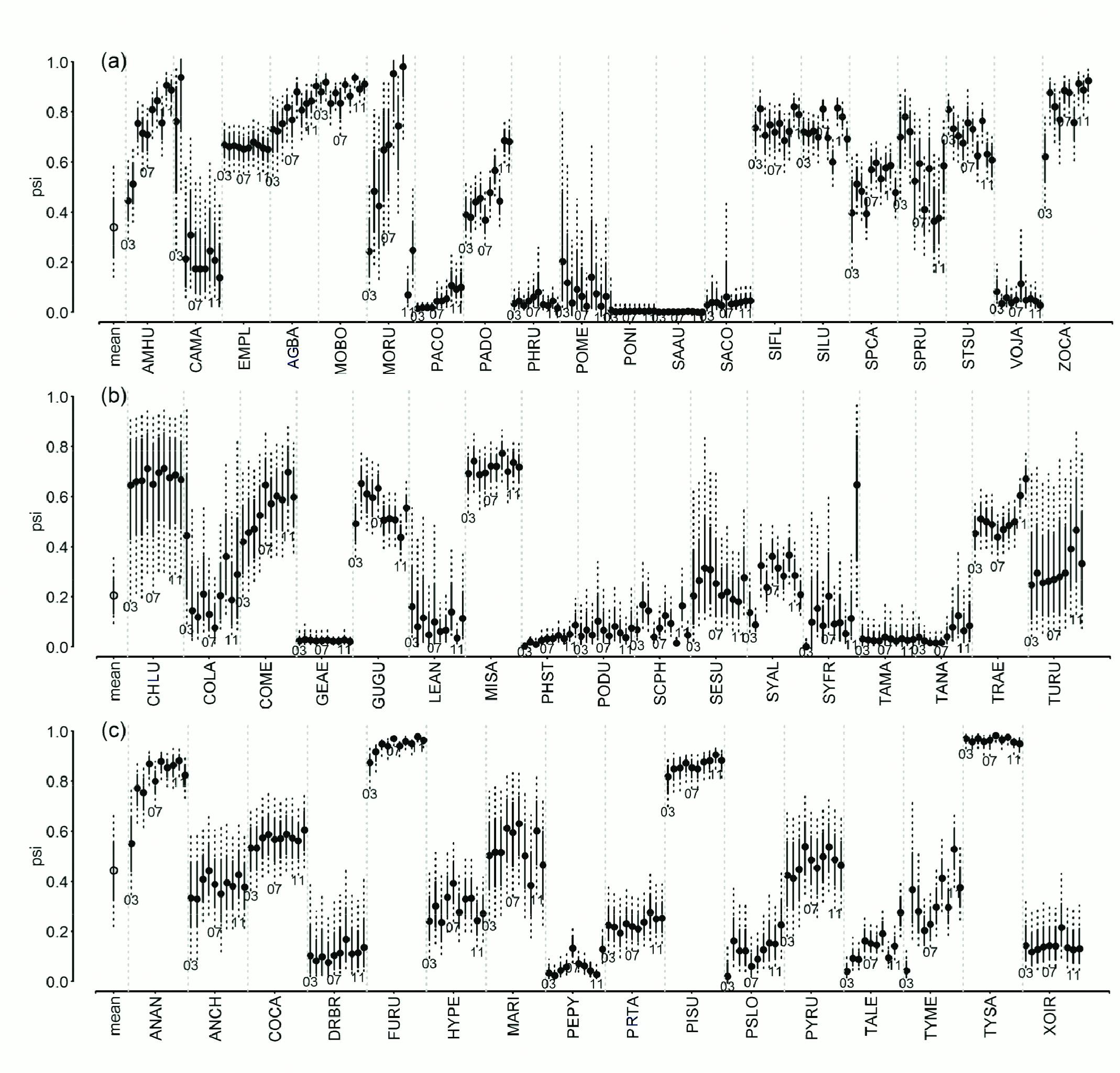

Supplement: S7 Fig — (a) Granivores; (b) insectivores mostly associated with folliage; (c) other insectivores. For details of species names and guilds, see S1 Table. (TIF) [file pone.0130874.s016.tif]

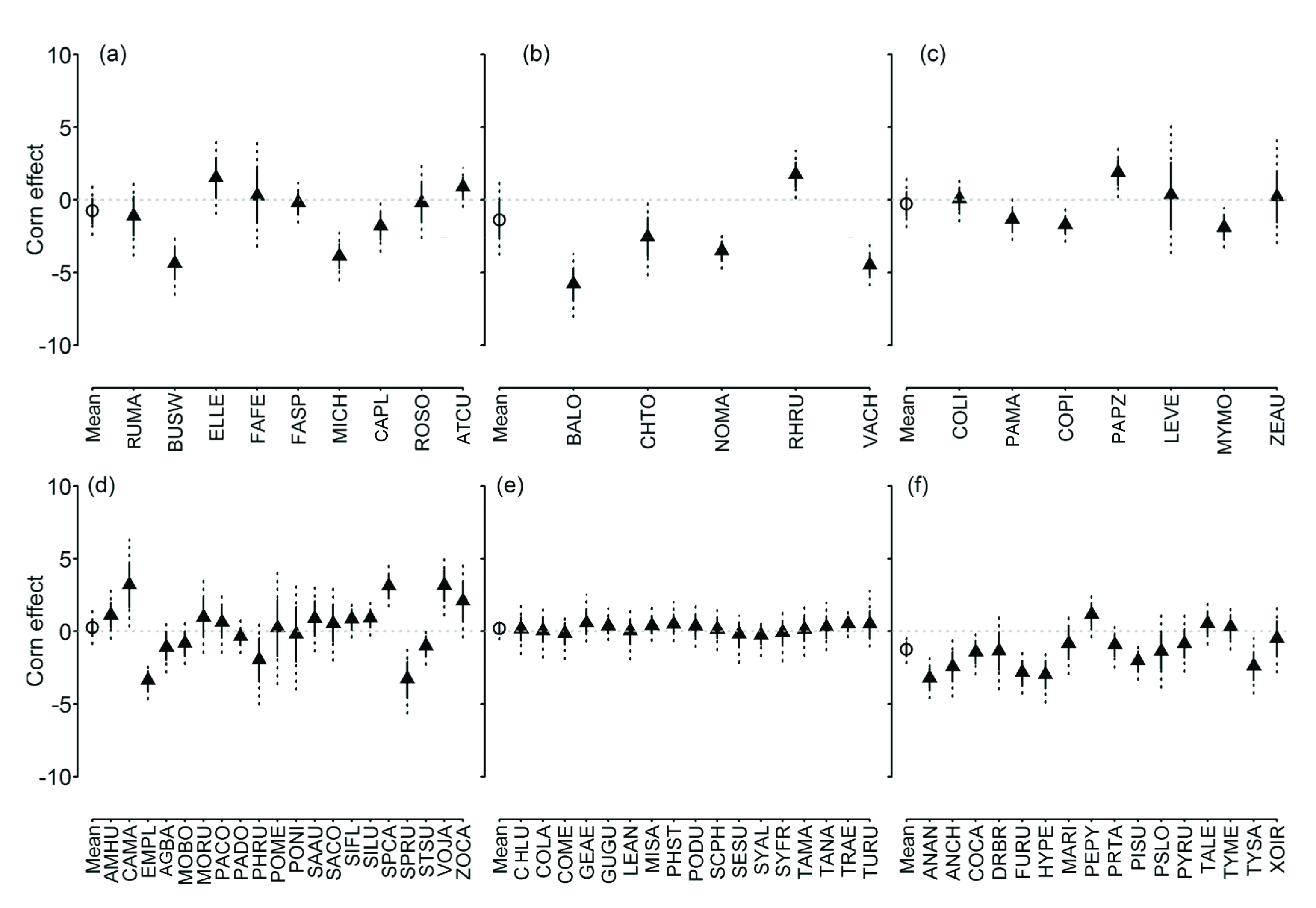

Supplement: S8 Fig — (a) Raptors; (b) ground omnivores and herbivores; (c) ground granivores; (d) other granivores; (e) insectivores mostly associated with folliage; (f) other insectivores. For details of species names and guilds, see S1 Table. (TIF) [file pone.0130874.s017.tif]

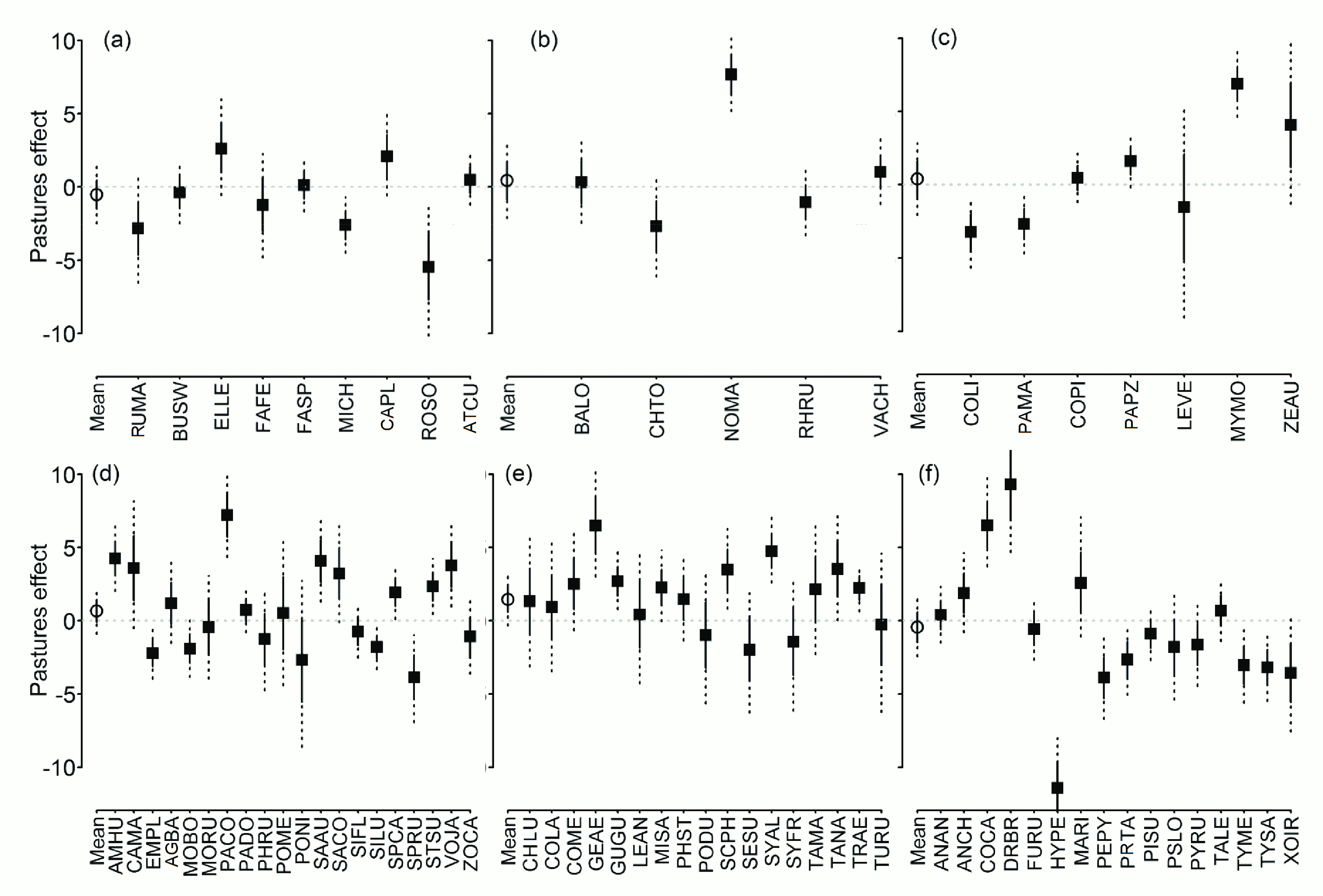

Supplement: S9 Fig — (a) Raptors; (b) ground omnivores and herbivores; (c) ground granivores; (d) other granivores; (e) insectivores mostly associated with folliage; (f) other insectivores. For details of species names and guilds, see S1 Table. (TIF) [file pone.0130874.s018.tif]
