# Supplementary material for: Multi-Season Regional Analysis of Multi-Species Occupancy: Implications for Bird Conservation in Agricultural Lands in East-Central Argentina
Source: PLoS One. 2015 Jun 18;10(6):e0130874. doi: 10.1371/journal.pone.0130874 (PMC4472512; doi:10.1371/journal.pone.0130874)
Supplement: S1 Table — Groups for analyses purposes and guilds are indicated: raptors (RAP), ground omnivores and herbivores (OMN), ground granivores (GRA2), other granivores (GRA), insectivores mostly associated with folliage (INS1) and other insectivores (INS2) [43,48,49]. (DOCX) [file pone.0130874.s019.docx]

| Group (guild)^1^ | Code | Family | Scientific name | Common name |
| --- | --- | --- | --- | --- |
| RAP (1) | RUMA | Accipitridae | *Rupornis magnirostris* | Roadside Hawk |
| RAP (1) | BUSW | Accipitridae | *Buteo swainsoni* | Swainson’s Hawk |
| RAP (1) | ELLE | Accipitridae | *Elanus leucurus* | White-tailed Kite |
| RAP (1) | ROSO | Accipitridae | *Rostrhamus sociabilis* | Snail Kite |
| RAP (1) | FAFE | Falconidae | *Falco femoralis* | Aplomado Falcon |
| RAP (1) | FASP | Falconidae | *Falco sparverius* | American Kestrel |
| RAP (1) | MICH | Falconidae | *Milvago chimango* | Chimango Caracara |
| RAP (1) | CAPL | Falconidae | *Caracara plancus* | Southern Crested Caracara |
| RAP (2) | ATCU | Strigidae | *Athene cunicularia* | Burrowing Owl |
| OMN (3) | CHTO | Anhimidae | *Chauna torquata* | Southern Screamer |
| OMN (3) | VACH | Charadriidae | *Vanellus chilensis* | Southern Lapwing |
| OMN (3) | BALO | Scolopacidae | *Bartramia longicauda* | Upland Sandpiper |
| OMN (3) | RHRU | Tinamidae | *Rhynchotus rufescens* | Red-winged Tinamou |
| OMN (3) | NOMA | Tinamidae | *Nothura maculosa* | Spotted Nothura |
| GRA2 (4) | COLI | Columbidae | *Columba livia* | Rock Dove |
| GRA2 (4) | PAMA | Columbidae | *Patagioenas maculosa* | Spot-winged Pigeon |
| GRA2 (4) | PAPZ | Columbidae | *Patagioenas picazuro* | Picazuro Pigeon |
| GRA2 (4) | COPI | Columbidae | *Columbina picui* | Picui Ground Dove |
| GRA2 (4) | LEVE | Columbidae | *Leptotila verreauxi* | White-tipped Dove |
| GRA2 (4) | ZEAU | Columbidae | *Zenaida auriculata* | Eared Dove |
| GRA2 (5) | MYMO | Psittacidae | *Myiopsitta monachus* | Monk Parakeet |
| GRA (5) | SAAU | Cardinalidae | *Saltator aurantiirostris* | Golden-billed Saltator |
| GRA (5) | SACO | Cardinalidae | *Saltator coerulescens* | Greyish Saltator |
| GRA (5) | PHRU | Cotingidae | *Phytotoma rutila* | White-tipped Plantcutter |
| GRA (5) | AMHU | Emberizidae | *Ammodramus humeralis* | Grassland Sparrow |
| GRA (5) | EMPL | Emberizidae | *Embernagra platensis* | Great Pampa Finch |
| GRA (5) | PACO | Emberizidae | *Paroaria coronata* | Red-crested Cardinal |
| GRA (5) | POME | Emberizidae | *Poospiza melanoleuca* | Black-capped Warbling-Finch |
| GRA (5) | PONI | Emberizidae | *Poospiza nigrorufa* | Black-and-rufousWarbling-Finch |
| GRA (5) | SIFL | Emberizidae | *Sicalis flaveola* | Saffron Finch |
| GRA (5) | SILU | Emberizidae | *Sicalis luteola* | Grassland Yellow-Finch |
| GRA (5) | VOJA | Emberizidae | *Volatinia jacarina* | Blue-black Grassquit |
| GRA (5) | ZOCA | Emberizidae | *Zonotrichia capensis* | Rufous-collared Sparrow |
| GRA (5) | CAMA | Fringillidae | *Carduelis magellanica* | Hooded Siskin |
| GRA (5,9) | AGBA | Icteridae | *Agelaioides badius* | Bay-winged Cowbird |
| GRA (5,9) | STSU | Icteridae | *Sturnella supercilliaris* | White-browed Blackbird |
| GRA (5,9) | MOBO | Icteridae | *Molothrus bonariensis* | Shiny Cowbird |
| GRA (5,9) | MORU | Icteridae | *Molothrus rufoaxillaris* | Screaming Cowbird |
| GRA (5) | PADO | Passeridae | *Passer domesticus* | House Sparrow |
| GRA (5) | SPCA | Thraupidae | *Sporophila caerulecens* | Double-collared Seedeater |
| GRA (5) | SPRU | Thraupidae | *Sporophila ruficollis* | Dark-throated Seedeater |
| INS1 (6) | PHST | Furnariidae | *Phacellodomus striaticollis* | Freckle-breasted Thornbird |
| INS1 (6) | SCPH | Furnariidae | *Schoeniophylax phryganophila* | Chotoy Spinetail |
| INS1 (6) | SYAL | Furnariidae | *Synallaxis albescens* | Pale-breasted Spinetail |
| INS1 (6) | SYFR | Furnariidae | *Synallaxis frontalis* | Sooty-fronted Spinetail |
| INS1 (6) | GEAE | Parulidae | *Geothlypis aequinoctialis* | Masked Yellowthroat |
| INS1 (6) | PODU | Polioptilidae | *Polioptila dumicola* | Masked Gnatcatcher |
| INS1 (6) | TAMA | Thamnophilidae | *Taraba major* | Great Antshrike |
| INS1 (6) | TRAE | Troglodytidae | *Troglodytes aedon* | House Wren |
| INS1 (6) | SESU | Tyrannidae | *Serpophaga subcristata* | White-crested Tyrannulet |
| INS1 (7) | CHLU | Trochilidae | *Chlorostilbon lucidus* | Glittering-bellied Emerald |
| INS1 (8) | LEAN | Dendrocolaptidae | *Lepidocolaptes angustirostris* | Narrow-billed Woodcreeper |
| INS1 (8) | COME | Picidae | *Colaptes melanochloros* | Green-barred Woodpecker |
| INS1 (6) | COLA | Cuculidae | *Coccyzus melacoryphus* | Dark-billed Cuckoo |
| INS1 (9) | GUGU | Cuculidae | *Guira guira* | Guira Cuckoo |
| INS1 (6) | TANA | Cuculidae | *Tapera naevia* | Striped Cuckoo |
| INS1 (9) | MISA | Mimidae | *Mimus saturninus* | Chalk-browed Mockingbird |
| INS1 (9) | TURU | Turdidae | *Turdus rufiventris* | Rufous-bellied Thrush |
| INS2 (10) | DRBR | Dendrocolaptidae | *Drymornis bridgesii* | Scimitar-billed Woodcreeper |
| INS2 (10) | ANAN | Furnariidae | *Anumbius annumbi* | Firewood-gatherer |
| INS2 (10) | FURU | Furnariidae | *Furnarius rufus* | Rufous Hornero |
| INS2 (10) | PSLO | Furnariidae | *Pseudoseisura lophotes* | Brown Cacholote |
| INS2 (10) | ANCH | Motacillidae | *Anthus lutescens* | Yellowish Pipit |
| INS2 (11) | HYPE | Tyrannidae | *Hymenops perspicillatus* | Spectacled Tyrant |
| INS2 (10) | MARI | Tyrannidae | *Machetornis rixosa* | Cattle Tyrant |
| INS2 (11) | PISU | Tyrannidae | *Pitangus sulphuratus* | Great Kiskadee |
| INS2 (11) | PYRU | Tyrannidae | *Pyrocephalus rubinus* | Vermilion Flycatcher |
| INS2 (11) | TYME | Tyrannidae | *Tyrannus melancholicus* | Tropical Kingbird |
| INS2 (11) | TYSA | Tyrannidae | *Tyrannus savana* | Fork-tailed Flycatcher |
| INS2 (11) | XOIR | Tyrannidae | *Xolmis irupero* | White Monjita |
| INS2 (12) | PEPY | Hirundinidae | *Petrochelidon pyrrhonota* | Cliff Swallow |
| INS2 (12) | PRTA | Hirundinidae | *Progne tapera* | Brown-chested Martin |
| INS2 (12) | TALE | Hirundinidae | *Tachycineta leucorrhoa* | White-rumped Swallow |
| INS2 (8) | COCA | Picidae | *Colaptes campestris* | Campo Flicker |

*Guilds:* 1= Diurnal raptors; 2= Nocturnal raptors; 3= Ground omnivores; 4= Ground granivores; 5= Granivore foliage gleaners; 6= Insectivorous foliage gleaners; 7= Insectivorous/nectarivorous; 8= Bark insectivores; 9= Ground and foliage omnivores; 10= Ground insectivores; 11 = Insectivorous salliers; 12 = Insectivorous Aerial foragers.
