# Supplementary material for: Multi-Season Regional Analysis of Multi-Species Occupancy: Implications for Bird Conservation in Agricultural Lands in East-Central Argentina
Source: PLoS One. 2015 Jun 18;10(6):e0130874. doi: 10.1371/journal.pone.0130874 (PMC4472512; doi:10.1371/journal.pone.0130874)
Supplement: S2 Table — (DOCX) [file pone.0130874.s020.docx]

| Group | Species | logit(*β_forest_*) | LBCI | | UBCI |
| --- | --- | --- | --- | --- | --- |
| RAP | RUMA | 3.123 | 2.451 | 3.795 | |
| RAP | BUSW | 0.189 | -0.508 | 0.888 | |
| RAP | ELLE | 0.266 | -0.604 | 1.078 | |
| RAP | FAFE | -1.967 | -3.071 | -0.967 | |
| RAP | FASP | -1.322 | -1.778 | -0.889 | |
| RAP | MICH | -1.298 | -1.556 | -1.041 | |
| RAP | CAPL | -0.497 | -0.785 | -0.224 | |
| RAP | ROSO | -0.839 | -1.966 | 0.184 | |
| RAP | ATCU | -4.324 | -4.784 | -3.874 | |
| OMN | BALO | -2.523 | -3.433 | -1.877 | |
| OMN | CHTO | -2.227 | -3.314 | -1.323 | |
| OMN | NOMA | -2.225 | -2.509 | -1.936 | |
| OMN | RHRU | -1.749 | -2.206 | -1.212 | |
| OMN | VACH | -2.045 | -2.227 | -1.858 | |
| GRA2 | COLI | -1.388 | -2.275 | -0.505 | |
| GRA2 | PAMA | 1.477 | 1.281 | 1.679 | |
| GRA2 | COPI | 2.658 | 2.455 | 2.869 | |
| GRA2 | PAPZ | 1.494 | 1.239 | 1.755 | |
| GRA2 | LEVE | 2.555 | 2.170 | 2.935 | |
| GRA2 | MYMO | 1.461 | 1.287 | 1.639 | |
| GRA2 | ZEAU | 1.851 | 1.643 | 2.059 | |
| GRA | AMHU | -4.052 | -4.326 | -3.783 | |
| GRA | CAMA | -0.538 | -1.293 | 0.176 | |
| GRA | EMPL | -2.359 | -2.878 | -1.851 | |
| GRA | AGBA | 2.405 | 2.130 | 2.672 | |
| GRA | MOBO | 1.008 | 0.784 | 1.231 | |
| GRA | MORU | 0.696 | 0.261 | 1.106 | |
| GRA | PACO | 0.445 | 0.155 | 0.727 | |
| GRA | PADO | 0.912 | 0.593 | 1.230 | |
| GRA | PHRU | 3.450 | 2.628 | 4.308 | |
| GRA | POME | 1.210 | 0.620 | 1.776 | |
| GRA | PONI | 0.000 | -0.630 | 0.599 | |
| GRA | SAAU | 1.458 | 1.208 | 1.712 | |
| GRA | SACO | 2.019 | 1.242 | 2.789 | |
| GRA | SIFL | 1.107 | 0.871 | 1.345 | |
| GRA | SILU | -2.255 | -2.529 | -1.977 | |
| GRA | SPCA | -0.442 | -0.639 | -0.251 | |
| GRA | SPRU | -3.172 | -3.892 | -2.484 | |
| GRA | STSU | -2.383 | -2.728 | -2.047 | |
| GRA | VOJA | -1.488 | -2.104 | -0.881 | |
| GRA | ZOCA | -1.078 | -1.240 | -0.920 | |
| INS1 | CHLU | 0.637 | 0.033 | 1.207 | |
| INS1 | COLA | 1.699 | 1.167 | 2.279 | |
| INS1 | COME | 1.705 | 1.352 | 2.067 | |
| INS1 | GEAE | 0.813 | 0.354 | 1.254 | |
| INS1 | GUGU | 0.682 | 0.492 | 0.880 | |
| INS1 | LEAN | 1.997 | 1.431 | 2.569 | |
| INS1 | MISA | 0.821 | 0.588 | 1.047 | |
| INS1 | PHST | 0.387 | -0.114 | 0.863 | |
| INS1 | PODU | 1.810 | 1.408 | 2.210 | |
| INS1 | SCPH | 0.271 | -0.187 | 0.712 | |
| INS1 | SESU | 2.205 | 1.553 | 2.931 | |
| INS1 | SYAL | 1.201 | 0.858 | 1.535 | |
| INS1 | SYFR | 1.689 | 1.115 | 2.245 | |
| INS1 | TAMA | 2.024 | 1.592 | 2.476 | |
| INS1 | TANA | 1.618 | 1.238 | 2.004 | |
| INS1 | TRAE | 0.957 | 0.736 | 1.181 | |
| INS1 | TURU | 1.146 | 0.671 | 1.622 | |
| INS2 | ANAN | 0.346 | -0.072 | 0.761 | |
| INS2 | ANCH | -3.009 | -4.686 | -1.516 | |
| INS2 | COCA | -0.565 | -0.891 | -0.245 | |
| INS2 | DRBR | 1.061 | 0.526 | 1.594 | |
| INS2 | FURU | 2.514 | 2.326 | 2.702 | |
| INS2 | HYPE | -2.095 | -3.424 | -0.804 | |
| INS2 | MARI | 0.593 | 0.063 | 1.111 | |
| INS2 | PEPY | -0.648 | -1.529 | 0.177 | |
| INS2 | PRTA | 0.194 | -0.047 | 0.425 | |
| INS2 | PISU | 1.799 | 1.598 | 2.007 | |
| INS2 | PSLO | 1.486 | 1.120 | 1.845 | |
| INS2 | PYRU | 2.423 | 1.706 | 3.125 | |
| INS2 | TALE | -0.760 | -1.508 | -0.070 | |
| INS2 | TYME | 2.584 | 2.171 | 3.003 | |
| INS2 | TYSA | 0.092 | -0.095 | 0.282 | |
| INS2 | XOIR | -0.995 | -1.445 | -0.561 | |

*Notes*: raptors (RAP), ground omnivores and herbivores (OMN), ground granivores (GRA2), other granivores (GRA), insectivores mostly associated with folliage (INS1) and other insectivores (INS2). For details of species names and guilds, see Table S2.
